# Parkinson’s disease, epilepsy, and amyotrophic lateral sclerosis—emerging role of AMPA and kainate subtypes of ionotropic glutamate receptors

**DOI:** 10.3389/fcell.2023.1252953

**Published:** 2023-10-24

**Authors:** Marina N. Vukolova, Laura Y. Yen, Margarita I. Khmyz, Alexander I. Sobolevsky, Maria V. Yelshanskaya

**Affiliations:** ^1^ Department of Pathophysiology, I.M. Sechenov First Moscow State Medical University (Sechenov University), Moscow, Russia; ^2^ Department of Biochemistry and Molecular Biophysics, Columbia University, New York, NY, United States; ^3^ Cellular and Molecular Physiology and Biophysics Graduate Program, Columbia University, New York, NY, United States; ^4^ N. V. Sklifosovsky Institute of Clinical Medicine, I.M. Sechenov First Moscow State Medical University (Sechenov University), Moscow, Russia

**Keywords:** glutamate receptors, AMPA, Parkinson’s disease, epilepsy, ALS, kainate receptors, 4-BCCA, antagonist

## Abstract

Ionotropic glutamate receptors (iGluRs) mediate the majority of excitatory neurotransmission and are implicated in various neurological disorders. In this review, we discuss the role of the two fastest iGluRs subtypes, namely, α-amino-3-hydroxy-5-methyl-4-isoxazolepropionic acid (AMPA) and kainate receptors, in the pathogenesis and treatment of Parkinson’s disease, epilepsy, and amyotrophic lateral sclerosis. Although both AMPA and kainate receptors represent promising therapeutic targets for the treatment of these diseases, many of their antagonists show adverse side effects. Further studies of factors affecting the selective subunit expression and trafficking of AMPA and kainate receptors, and a reasonable approach to their regulation by the recently identified novel compounds remain promising directions for pharmacological research.

## 1 Introduction

**FIGURE 1:**
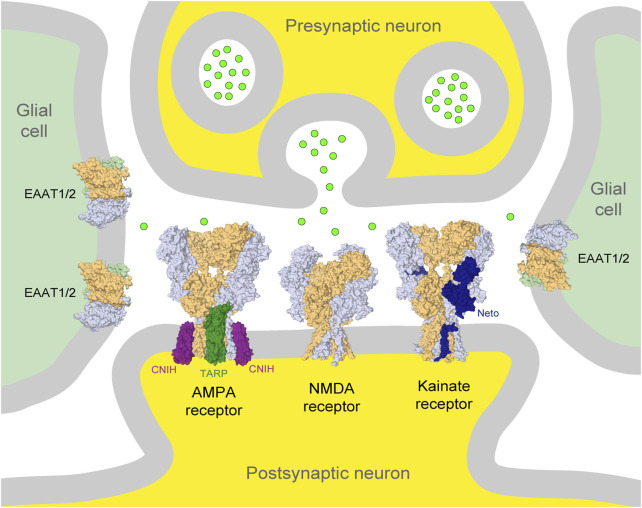
Glutamatergic synapse. Presynaptic and postsynaptic neuronal terminals are shown at the top and bottom in the center (yellow), respectively, and glial cells on the left and right (light green). Neurotransmitter glutamate released from the presynaptic terminus is illustrated by bright green circles. AMPA, NMDA, and kainate iGluRs in the postsynaptic membrane and glutamate transporters (EAAT1/2) in glial cells are illustrated by the corresponding molecular models in surface representation. Auxiliary subunits, TARP and CNIH, of the AMPA receptor are represented in dark green and purple, respectively, and Neto of the kainate receptor is represented in dark blue. The principal subunits of iGluRs are represented in light blue and beige, and glutamate transporters are represented in light blue, beige, and light green.

The chemical messenger glutamate mediates most excitatory neurotransmission in the mammalian central nervous system (CNS). Although the concentration of glutamate is strictly regulated in physiological conditions, its elevated levels in the synaptic cleft are the principal cause of neuronal death upon stroke and traumatic brain injury, as well as in neurodegenerative conditions (Roisen et al., 1982; Buchan et al., 1993; Couratier et al., 1993). Exposure of neuronal culture to excessive concentrations of glutamate results in rapid cell death (Choi et al., 1988; Olney, 1994) that can be prevented by blocking ionotropic glutamate receptors (iGluRs). iGluRs are cation-permeable glutamate-gated ion channels located predominantly in postsynaptic neuronal membranes (Figure 1). They play a key role in synaptic transmission in the CNS and are involved in synaptic plasticity and processes underlying learning and memory (Lynch, 2006). It is, therefore, not surprising that their dysregulation is associated with numerous pathophysiological conditions (Liu and Zukin, 2007; Traynelis et al., 2010; Paoletti et al., 2013; Parsons and Raymond, 2014; Hansen et al., 2021).

**FIGURE 2:**
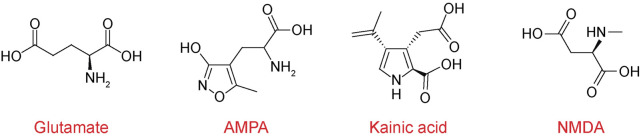
Chemical structures of iGluR agonists. Chemical structures of neurotransmitter glutamate and selective agonists AMPA (α-amino-3-hydroxy-5-methyl-4-isoxazolepropionic acid), kainate, and NMDA (N-methyl-d-aspartate).

iGluRs are divided into four functional classes: 1) α-amino-3-hydroxy-5-methyl-4-isoxazolepropionic acid (AMPA) receptors (GluA1-4 subunits), 2) kainate receptors (GluK1-5 subunits), 3) N-methyl-d-aspartate (NMDA) receptors (GluN1, GluN2A-D, and GluN3A-B subunits), and 4) GluD receptors, also known as δ receptors (GluD1 and GluD2 subunits). iGluRs were originally named based on their specific activators (Figure 2). AMPA and kainate receptors coassemble with different auxiliary subunits, such as the transmembrane AMPA receptor regulatory proteins (TARPs), cysteine-knot AMPA receptor modulating proteins (CKAMPs), neuropilin- and tolloid-like (Neto) proteins, and KRIP6 (a protein from the BTB/Kelch family) that display differential distribution throughout CNS and cause changes in receptor function and sensitivity to modulators (Figure 1) (Laezza et al., 2007; Gardinier et al., 2016; Kato et al., 2016; Maher et al., 2016; Twomey et al., 2019; Yu et al., 2020; Cull-Candy and Farrant, 2021; Hansen et al., 2021; Yelshanskaya and Sobolevsky, 2022).

It is generally accepted that neurodegeneration correlates negatively with synaptic plasticity, which is driven by iGluR function. Since the role of iGluRs in neurodegeneration is a very broad topic, we will only discuss the mechanisms of glutamatergic system dysregulation in the neurogenerative conditions of Parkinson’s disease (PD), epilepsy, and amyotrophic lateral sclerosis (ALS), particularly focusing on non-NMDA receptors, as this topic aligns most with the interests of the authors of this review. We try to be cautious in not oversimplifying the role of iGluRs as they represent a fraction of the larger dynamic ensemble of neuronal receptors involved in synaptic plasticity, which are continuously regulated by new protein biogenesis and trafficking between synaptic and extrasynaptic pools.

## 2 Parkinson’s disease

PD is the second most common progressive neurodegenerative disorder after Alzheimer’s disease. PD affects 1%–3% of individuals age 65 years or older (Dorsey et al., 2018), and in 3%–5% of cases, individuals can display symptoms previously, before the age of 40 (Golbe, 1991). The estimated number of people with PD in 1990 was 2.5 million, which has more than double and reached 6.2 million by 2015 and is expected to double again and reach 12.9 million by 2040 (Elbaz et al., 2016; Dorsey and Bloem, 2018). PD has been known to mankind since ancient times. In the Indian medical system of Ayurveda (5000 BC), it was called kampavata (“kampa” means tremor in Sanskrit). PD symptoms were also described in the Chinese medical text “Nei-Jing” (500 BC). In Western medicine, the disease was named after Doctor James Parkinson, whose “*Essay on the Shaking Palsy”* (1817) has long been considered the foundational text about PD (Hurwitz, 2014; Maiti et al., 2017). PD is associated with substantial disability and negative impact on the quality of life, causing characteristic motor symptoms of tremor, bradykinesia, and postural instability (Dorsey et al., 2007). These symptoms are coupled to the loss or degeneration of dopaminergic (dopamine-producing) neurons and development of Lewy Bodies (a pathologic hallmark) in the *substantia nigra* region of the brain and their axonal projections to the *striatum* (Maiti et al., 2017). The loss of neurons is followed by the death of astrocytes, which then increases the amount and activation of microglia in the *substantia nigra pars compacta (SNpc)* (Jankovic, 2008). Clinical symptoms do not appear immediately; they became apparent at the point when approximately half of the cells are destroyed and the disease has already progressed to an advanced stage (Cheng et al., 2011).

Although the causes and driving forces of PD are not well understood, several disease risk factors have been linked to the degeneration of the dopaminergic neurons, including genetics, obesity, and neuroinflammation caused by various environment factors (i.e., exposure to industrial chemicals, pesticides like rotenone, herbicide paraquat, and heavy metals), gut health (Tanner et al., 2011; Bjorklund et al., 2018; Fan et al., 2020; De Miranda et al., 2022), or a combination of them. Thus, recent experiments on mice suggested a synergy between the diet (lectins ingestion), gut health, and environmental toxins in the development of PD (Anselmi et al., 2018).

Currently, there is no cure for PD; the main treatment is symptomatic, and pharmacological interventions have various limitations and side effects. The development of effective preventive or protective therapies is limited by our knowledge of the causes and mechanisms by which neurons die in PD. Which components of neurotransmission that are known to be involved in the pathogenesis of PD play a primary or secondary role in neurodegeneration is not yet well understood because imbalances in the dopamine-releasing system cause further disturbances and imbalances of other components, i.e., acetylcholine/dopamine/glutamate neurotransmission. Although glutamatergic signaling increases and stimulates the release of dopamine through surviving dopaminergic neurons in the SNpc as a compensatory mechanism, increasing glutamate concentrations and excessive activation of glutamate receptors could be a “critical strike” to dopaminergic neurons in PD patients as well (Wang et al., 2020). The approved PD treatments include the use of dopamine receptor agonists (for example, L-DOPA), dopamimetic drugs to relieve the symptoms of impaired motor function (Lutsenko et al., 2003), and deep brain stimulation techniques (Malek, 2019). Although these forms of treatment may partially ameliorate the motor dysfunctions of PD patients, they do not slow the disease progression. Moreover, prolonged therapy frequently leads to the development of motor complications, known as L-DOPA-induced dyskinesia (LID), and dementia. In turn, motor dysfunction is linked to impaired AMPA receptor plasticity (Jurado, 2017; Zhang et al., 2019c; Zhang and Bramham, 2020). Indeed, compared to healthy individuals, synapses of PD patients with motor disturbances accumulate excessive glutamate (Mironova et al., 2018). It is known that elevated oxidative stress causes mutations in glutamate transporters and thus leads to elevated glutamate concentrations in the synaptic cleft (Hoye et al., 2008). The resulting failure to quickly clear synaptic glutamate triggers repetitive action potentials, an increase in calcium influx, and endoplasmic reticulum (ER) and mitochondrial stress due to the overwhelmed ability to calcium storage (Becker et al., 2017). In addition, the upregulation of AMPA receptors in the *lateral putamen* was observed in advanced PD patients experiencing LID when compared to patients without motor complications (Calon et al., 2003). Accordingly, there is an enormous need to design therapeutics to stop the progression of PD (Zhang and Bramham, 2020; O'Neill et al., 2004; Hughes et al., 1992).

Early detection of PD is crucial for effective neurodegenerative disease interventions. Correspondingly, many researchers focus on identifying genetic factors that increase the risk of disease. Mutations in at least 20 genes have been recognized as causes of familial parkinsonism, each providing a snapshot into the molecular basis of neurodegeneration. Over 90 genetic risk loci for the more common sporadic form of PD have already been identified (Blauwendraat et al., 2020). Although it is more challenging to unravel the precise biological processes disrupted in these genetic variants, the disease-associated genes begin to coalesce into common pathways, including the dysregulation of mitochondrial homeostasis, impaired cell death machinery, inflammatory signaling, intracellular trafficking, and endosomal–lysosomal dysfunction (Tolosa et al., 2021). Genetic predisposition for the early onset of PD was determined for patients with mutations in one of the dominant genes, namely, *SNCA*, *LRRK2*, *GBA*, and *VPS35* or recessive genes, namely, *Parkin*, *Pink1*, and *DJ1* (Post et al., 2020).

Effective strategies for the treatment of PD include normalizing glutamate homeostasis, reducing oxidative stress, and attenuating glial activation (Martin-Moreno et al., 2011; Fernandez-Ruiz et al., 2013; Bhunia et al., 2022). During PD, the mitochondrial Ca^2+^-buffering system in *substantia nigra* neurons was shown to be impaired and led to Ca^2+^-induced excitotoxicity (Hoye et al., 2008; Hurley et al., 2013). One of the strategies in the treatment of PD is limiting the excessive influx of Ca^2+^ into neurons, including the direct blocking of iGluRs (O'Neill and Witkin, 2007; Jayakar and Dikshit, 2004; Calabrese et al., 2017). In primates, a decrease in LID was observed due to the NMDA receptor channel block (Zuddas et al., 1992; Papa and Chase, 1996). Although NMDA receptor antagonists showed a positive effect against LID both in mice and primates, clinical trials have not yet achieved the desired effects in humans (Duty, 2012). In contrast to NMDA receptors, which are usually inactive at the resting membrane potential due to the channel block by Mg^2+^ (Mayer et al., 1984; Nowak et al., 1984; Sobolevsky and Yelshansky, 2000), AMPA and kainate receptors are not blocked by extracellular cations and some of them (depending on subunit composition) allow Ca^2+^ entry into the cell upon activation. AMPA and kainate receptors are typically expressed as heterotetramers, and those assemblies that contain edited GluA2 (for AMPA receptors) or GluK1/2 (for kainate receptors) subunits are calcium-impermeable, while other combinations are calcium-permeable. Due to their high permeability to Ca^2+^ (and as well to Zn^2+^) ions, the latter becomes the important target for pharmaceutical intervention (Hansen et al., 2021) (Tables 1, 2). The antagonists of calcium-permeable AMPA receptors were shown to slow the development of LID and reduce the progression of dyskinesia (Kobylecki et al., 2013). It was also shown that the increase in permeability of the *substantia nigra* neuronal membranes to extracellular zinc leads to the death of nigrostriatal dopaminergic neurons (Tamano et al., 2018). *In vivo* experiments on rats showed that the injection of the agonist AMPA into the area of spiny projection neurons caused a rapid increase in the intracellular Zn^2+^ ions and loss of nigrostriatal dopaminergic neurons weeks later. This increase was blocked by the coinjection of intracellular Zn^2+^ chelators ZnAF-2DA and TPEN, suggesting that the AMPA-induced movement disorder is also a result of intracellular Zn^2+^ dysregulation (Tamano et al., 2018). Therefore, the regulation of Ca^2+^- and Zn^2+^-permeable GluR2-lacking AMPA receptors appears to be particularly important for the treatment of PD.

**TABLE 1:** AMPA and Kainate receptor antagonists in the treatment of patients with Parkinson’s disease, Epilepsy, ALS.

Antagonist	Structure	Receptor	Disease	Patients	Dose, mg	Effect	Secondary outcome	First authors/ year
**Perampanel**	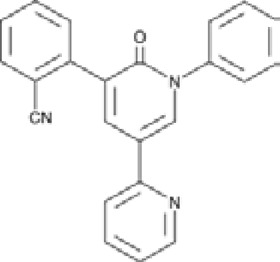	AMPA+KA	**Parkinson’s disease**	With a diagnosis of idiopathic PD, who were on optimized L-dopa therapy	0.5, 1, 2	Was well tolerated and safe, but failed to achieve statistical significance	ADE	Eggert et al. (2010)
				With levodopa-treated	2, 4	Failed to significantly improve motor symptoms versus placebo	No effect on the duration or disability of levodopa-induced dyskinesia	Lees et al. (2012)
				With a diagnosis of idiopathic PD, who were on optimized L-dopa therapy	4	Was generally well tolerated, was not superior to placebo on any efficacy end point	ADE	Rascol et al. (2012)
			**Epilepsy**	With partial seizures despite receiving	2, 4, 8, 12	Reduced partial seizure frequency and improved rates	ADE	Steinhoff et al. (2013)
				In status epilepticus (SE), refractory SE (RSE), super-refractory SE (SRSE)	2, 4, 8, 12, 16, 24, 32, 36	The efficacy in the treatment of RSE, SRSE	ADE	Lim et al. (2021)
				With temporal lobe and focal epilepsy	2 - 12	No significant difference	ADE	Mammi et al. (2022)
			**ALS**	Sporadic or familial possible/probable/definite ALS	2, 8	Its poor tolerability	ADE	Hotait et al. (2021)
				Clinically definite ALS	4, 8	Effects the physiology of the upper motor neurons	ADE	Oskarsson et al. (2021)
				Clinically definite ALS	4, 8	Significant decline in ALSFRS-R score and worsening of the bulbar subscore	Disease progression, ADE	Aizawa et al. (2022)
**Talampanel (GYKI537773, LY300164)**	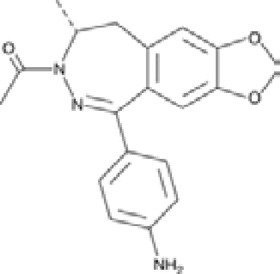	AMPA	**Epilepsy**	With intractable epilepsy	35, 75	No evidence that talampanel increased or decreased seizure frequency or changed the type of seizure	ADE	Langan et al. (2003)
				With refractory partial seizures	25, 50, 60, 75	Reduction in reducing seizure frequency - caused a dose-dependent increase in resting and active motor thresholds without effects on intra-cortical inhibition or facilitation	ADE	Bialer et al. (2002), Bialer et al. (2004), Bialer et al. (2007)
			**ALS**	With definite or probable ALS	20, 50	Decline in muscle strength and ALSFRS	ADE	Pascuzzi et al. (2010)
**NS1209**	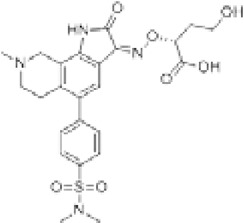	AMPA	**Epilepsy**	With convulsive or non-convulsive RSE	4, 75	No statistically significant difference found	ADE	Sabers et al. (2013)
**Selurampanel (BGG492)**	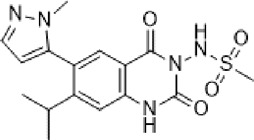	AMPA+KA	**Epilepsy**	With photosensitive epilepsy	15, 50, 100	Reduction of the SPR, complete suppression of the PPR	ADE	Faught (2014)
				With epilepsy and a generalized epileptiform electroencephalography response to intermittent photic stimulation	50, 100	Reduction of SPR range of at least three steps	ADE	Kasteleijn-Nolst Trenitet et al. (2015)
				With partial-onset seizures	100, 150	Reduction in total partial seizure frequency	ADE	Elger et al. (2017)

Abbreviations: ADE, adverse drug events; Perampanel, 5'-(2-cyanophenyl)-1'-phenyl-2,3'-bipyridinyl-6'(1H)-one; Talampanel (GYKI537773 and LY300164), (8R)-7-Acetyl-5-(4-aminophenyl)-8,9-dihydro-8-methyl-7H-1,3-dioxolo[4,5-h][2,3]benzodiazepine; NS1209, (RS)-NS 1209, 2-[[[5-[4-[(Dimethylamino)sulfonyl]phenyl]-1,2,6,7,8,9-hexahydro-8-methyl-2-oxo-3H-pyrrolo[3,2-h]isoquinolin-3-ylidene]amino]oxy]-4-hydroxybutanoic acid; Selurampanel (BGG492), N-[7-Isopropyl-6-(2-methylpyrazol-3-yl)-2,4-dioxo-1H-quinazolin-3-yl]methanesulfonamide; ALS, Amyotrophic lateral sclerosis; ALSFRS-R, Amyotrophic lateral sclerosis functional rating scale revised; SPR, the standardized PPR range; PPR, the photoparoxysmal response.

**TABLE 2:** The role of AMPA and Kainate receptor antagonists in model animals of Parkinson’s disease, Epilepsy, ALS.

Antagonist	Structure	Receptor	Disease	Model disease	Effect	First authors/year
**Perampanel**	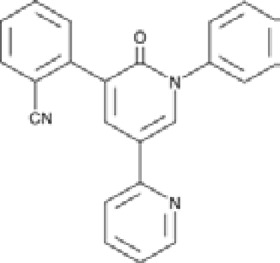	AMPA+KA	**Parkinson’s disease**	C57BL/6J male mice, mouse primary hippocampal neurons, an α-synuclein preformed fibril-injected mouse model	Inhibited the neuronal uptake of α-syn PFFs via macropinocytosis and decreased the development of α-synuclein pathology in primary neurons	Ueda et al. (2021)
			**Epilepsy**	Kindled rat, audiogenic, MES- and scMet-induced mouse seizure models	Demonstrated potent anticonvulsant activity in these seizure models	Bialer et al. (2010)
				Primary cortical neurons, male Wistar rat, mouse seizure models: audiogenic; 6 Hz-, MES- and PTZ-induced seizures	-Inhibited 6 Hz-induced, AMPA-induced increases in [Ca^2+^]_i_ -Protective effects against audiogenic, MES-induced, and PTZ-induced seizures	Hanada et al. (2011)
				Male ddY mice and Sprague-Dawley rats, mouse AMPA-induced seizure model	Potent activity *in vitro* AMPA-induced Ca^2+^ influx assay and *in vivo* AMPA-induced seizure model	Hibi et al. (2012)
				Whole-cell voltage-clamp recording in cultured rat hippocampal neurons	-Concentration-dependent inhibition of AMPA receptor currents evoked by AMPA and KA -The extent of block of non-desensitizing KA-evoked currents -Does not influence AMPA receptor desensitization	Chen et al. (2014)
				Male Sprague-Dawley rats, SE induction, Morris water maze	-Reduced GluA1 expression and regulated GluA1 phosphorylations by multiple signaling molecules -Increased pCAMKII, pPKA ratios, and elevated pJNK and pPP2B ratios -Increased pERK1/2 ratio in epileptic animals	Kim et al. (2019)
				Neonatal male and female C57BL/6 mice, cell culture and transfection	-Inhibited both recombinant and neuronal KA, also heteromeric GluK1/GluK5 and GluK2/GluK5 KA -Inhibited mouse neuronal KARs containing GluK5 subunits and Neto proteins in nociceptive dorsal root ganglia neurons and hippocampal mossy fiber–CA3 pyramidal neuron synapses	Taniguchi et al. (2022)
			**ALS**	Homozygous (ADAR2flox/flox/ VAChT-Cre.Fast; AR2) and heterozygous (ADAR2flox/+/ VAChT-Cre. Fast; AR2H) conditional ADAR2 knockout mice	Prevented the death of motor neurons and improves of motor dysfunction by long-term administration	Akamatsu et al. (2016)
**Talampanel (GYKI537773 and LY300164)**	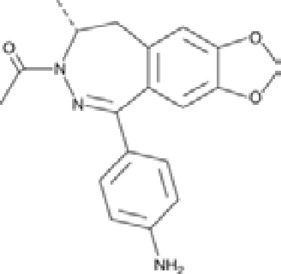	AMPA	**Parkinson’s disease**	Adult cynomolgus (Macaca fascicularis) monkeys, MPTP model	-Decreased L-dopa-induced dyskinesia -Potentiated the motor activating effects of low-dose L-dopa, increasing motor activity	Konitsiotis et al. (2000)
			**ALS**	Cell cultures of motor neurons and glial cells	-Dose-dependently inhibited the KA-induced motor neuron death -Blocked the KA-induced Co -uptake in motor neurons	Van Den Bosch et al. (2000)
				Hemizygous transgenic mice, expressing mutant human SOD1 with a G93A substitution, a C57BL/ 6JOlaHsd mice	Reduced elevated calcium level, but not restored, when the treatment was started presymptomatically	Paizs et al. (2011), Patai et al. (2017)
			**Epilepsy**	Wistar breeder rats, kainic acid-induced seizures	Delayed the commencement of tonic extension, but not status-induced by kainic acid	Dhir and Chavda (2016)
**NS1209**	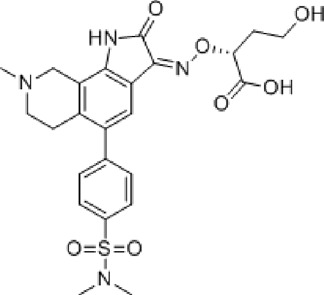	AMPA	**Epilepsy**	Male Harlan Sprague—Dawley (amygdala stimulation model), wistar rat (kainate model)	-Effectively discontinued electrically induced SE -Blocked the KA-induced SE -Neuroprotective effect against SE-induced hippocampal neurodegeneration	Pitkänen et al. (2007)
**Selurampanel (BGG-492)**	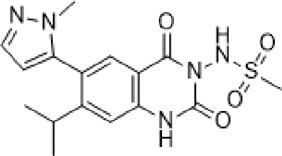	AMPA+KA	**Epilepsy**	Mice, the MES seizure model	Excellent potency against maximal electroshock seizure (MES)-induced generalized tonic–clonic seizures	Orain et al. (2016)
**UBP 310**	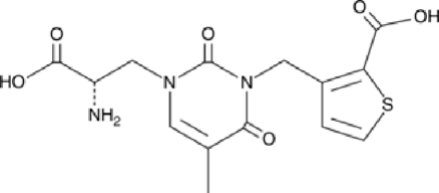	KA: GluK1, GluK2, AMPA: GluA2	**Parkinson’s disease**	C57BL/6 or GluK^1−/−^, GluK^2−/−,^ or GluK^3−/−^ male mice, unilateral 6-OHDA lesioning, acute MPTP mouse model of PD	-Did not attenuate cell loss in the midbrain induced by 6-OHDA toxicity -Increased survival dopaminergic and total neuron population in the substantia nigra but not in striatum in the acute MPTP mouse model	Stayte et al. (2020)
			**Epilepsy**	C57BL/6 wild-type and GluK1 and GluK3 knockout mice, male Wistar rat, electrophysiological recordings, TLE model	-Blocked postsynaptic KA at hippocampal mossy fiber (MF) CA3 synapses and in aberrant MF synapses in the epileptic hippocampus -Strongly reduced isolated KA-EPSCs recorded in DGCs of chronic epileptic rats, but fully spares AMPA EPSCs	Pinheiro et al. (2013)
				Male Wistar rat, hippocampal neuron-glial cell cultures, [Ca^2+^]_i_ imaging, whole-cell recordings	Decreased in the amplitude of the 1^st^ AP in PDSs and the amplitude of the oscillations of [Ca^2+^]_i_ occurring alongside the PDS cluster generation	Laryushkin et al. (2023)
**Tezampanel** **(LY293558)**	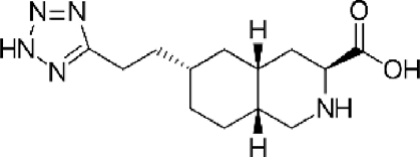	AMPA+KA	**Parkinson’s disease**	Male Sprague-Dawley rats, chronic L-dopa treatment, 6-OHDA lesions	Reversed the reduction in the duration of L-dopa response	Marin et al. (2001)
				Male Sprague-Dawley rats, 6-OHDA lesions	Reversed the increased overexpression of PPE mRNA induced by L-dopa treatment	Perier et al. (2002)
			**Epilepsy**	Male National Institutes of Health (NIH) Swiss mice, The kindling model (limbic Epilepsy)	Produced a dose-dependent suppression of the rate of development of behavioral kindled seizure activity and reduced the duration of the stimulation-induced electrographic afterdischarge	Rogawski et al. (2001)
				Male NSATM (CF#1^®^) mice, electroshock seizures, the 6-Hz test, the MES seizure model	Dose-dependent protection	Barton et al. (2003)
**NBQX**	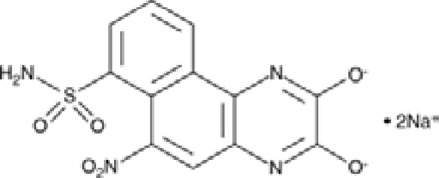	AMPA+KA	**Parkinson’s disease**	Male Sprague-Dawley rats, ascorbic acid or 6-OHDA behavioral tests	-Inhibited most aVTA dopaminergic neurons and DRN serotonergic neurons -Excited most pVTA dopaminergic neurons and MRN serotonergic neurons in the SNc sham and SNc lesion groups	Zhang et al. (2019a)
				C57BL/6J male mice, mouse primary hippocampal neurons, an α-synuclein preformed fibril-injected mouse model	Inhibited the neuronal uptake of α-syn PFFs via macropinocytosis and decreased the development of α-synuclein pathology in primary neurons	Ueda et al. (2021)
			**Epilepsy**	Male NSATM (CF#1^®^) mice, electroshock seizures, the 6-Hz test, the MES seizure model	Dose-dependent protection	Barton et al. (2003)
				Long-Evans rats experienced hypoxia-induced neonatal seizures	Attenuates later-life epileptic seizures and autistic-like social deficits following neonatal seizures	Lippman-Bell et al. (2013)
				Mouse model of mesial temporal lobe epilepsy	No effect on development or frequency of seizures was found in comparison to vehicle controls	Twele et al. (2015)
				Wistar rat hippocampal neuron-glial cell cultures, [Ca^2+^]_i_ imaging, whole-cell voltage-clamp recordings	Completely suppresses bicuculline-induced paroxysmal activity	Laryushkin et al. (2023)
			**ALS**	Wistar rat motor neuron cell cultures, transgenic SOD1 G93A mutant mice for familial ALS, [Ca^2+^]_i_ imaging, perforated patch clamp recordings	-Blocked KA-induced currents and concomitant changes in [Ca^2+^]_i_, -Prevented the KA-induced motor neuron death, -Prolonged survival G93A mutant mice	Van Damme et al. (2003)
**GYKI 53784** **(LY303070)**	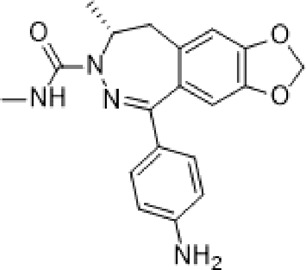	AMPA	**Epilepsy**	Vitro and vivo models of AMPARs-mediated excitotoxicity	A powerful neuroprotective agent, does not block the activation of KA	Ruel et al. (2002)
**GYKI 52466**	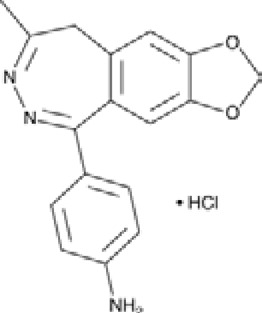	AMPA+KA	**Epilepsy**	Sprague-Dawley rat hippocampal neuron cell cultures, whole-cell voltage-clamp recordings	The block was voltage independent	Donevan and Rogawski (1993)
				Male National Institutes of Health (NIH) Swiss mice, The kindling model (limbic epilepsy)	Produced a dose-dependent suppression of the rate of development of behavioral kindled seizure activity and reduced the duration of the stimulation-induced electrographic afterdischarge	Rogawski et al. (2001)
				Male NSATM (CF#1^®^) mice, electroshock seizures, the 6-Hz test, the MES seizure model	Dose-dependent protection	Barton et al. (2003)
				The genetic absence epilepsy model of WAG/Rij rats	-A fast dose-dependent increase in the number and cumulative duration of SWD -Strong ataxia and immobility, decrease of active wakefulness and increase in deep slow wave sleep	Jakus et al. (2004)
**GYKI 53 655 (LY300168 hydrochloride)**	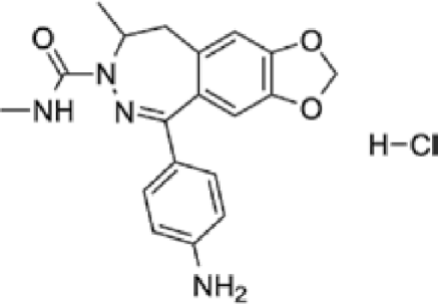	AMPA	**Epilepsy**	Male NSATM (CF#1^®^) mice, electroshock seizures, the 6-Hz test, the MES seizure model	Dose-dependent protection	Barton et al. (2003)
**LY377770**	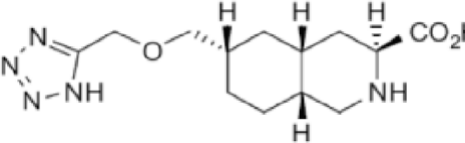	KA: GluK1,5	**Epilepsy**	Male NSATM (CF#1^®^) mice, electroshock seizures, the 6-Hz test, the MES seizure model	Dose-dependent protection	Barton et al. (2003)
				Human HEK293 cells, hippocampal slices obtained from Wistar rats, electrophysiology recordings	Blocked epileptiform activity in hippocampal slices and seizures in vivo induced by pilocarpine or electrical stimulation	Smolders et al. (2002)
**GYKI 53405** **(LY 293606)**	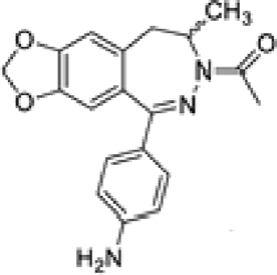	AMPA	**Epilepsy**	The genetic absence epilepsy model of WAG/Rij rats	Failed to affect any measure of SWD and vigilance	Jakus et al. (2004)
**GYKI53655** **(LY300168 hydrochloride)**	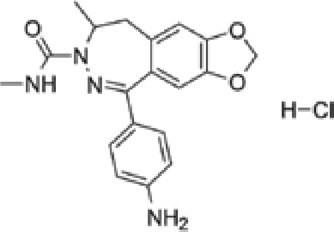	GluK3, Native KA, AMPA	**ALS**	Putative spinal motor neurons (mouse embryos), the patch-clamp technique	Completely blocked the KA-induced currents	Albo et al. (2004)
**CNQX**	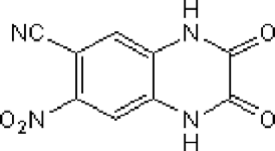	AMPA+KA	**ALS**	Cell cultures of motor neurons and glial cells	Blocked the motor neuron death	Van Den Bosch et al. (2000)
**RPR 119990**		AMPA	**ALS**	Transgenic mouse model of familial amyotrophic lateral sclerosis (SOD1-G93A)	-Displaced [3H]AMPA from rat cortex membranes -Potent anticonvulsant in the supramaximal electroshock -Prolong survival mice	Canton et al. (2001)
**29-fluoro (29-F) modified RNA aptamers FN58**		AMPA, KA, and NMDA	**ALS**	Male Homozygous the ADAR2flox/flox/ VAChT-Cre.Fast (AR2) knockout mice, a model of sporadic ALS	Reduced the progression of motor dysfunction, normalized TDP-43 mislocalization, and prevented death of motor neurons	Akamatsu et al. (2022)
**ACET**	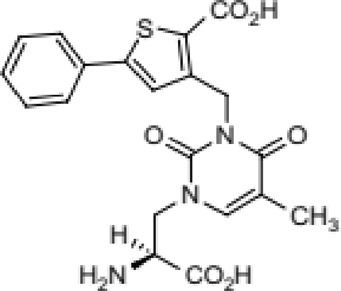	GluK1 (GluR5) KA	**Epilepsy**	Wistar rat hippocampal slices cell cultures	Significantly delayed developmental synchronization of the hippocampal CA3 network and generation of IEA	Atanasova et al. (2023)

Abbreviations: UBP310, (S)-1-(2-Amino-2-carboxyethyl)-3-(2-carboxy-thiophene-3-yl-methyl)-5-methylpyrimidine-2,4-dione; Tezampanel (LY293558), (3S,4aR,6R,8aR)-6-[2-(1H-1,2,3,4-tetrazol-5-yl)ethyl]-decahydroisoquinoline-3-carboxylic acid; Perampanel, 5'-(2-cyanophenyl)-1'-phenyl-2,3'-bipyridinyl-6'(1H)-one; Talampanel (GYKI537773 and LY300164), (8R)-7-Acetyl-5-(4-aminophenyl)-8,9-dihydro-8-methyl-7H-1,3-dioxolo[4,5-h][2,3]benzodiazepine; NBQX - 2,3-Dioxo-6-nitro-1,2,3,4-tetrahydrobenzo[f]quinoxaline-7-sulfonamide; GYKI53784 (LY303070), 1-(4-aminophenyl)-4-methyl-7,8-methylenedioxy4,5-dihydro-3-methylcarbamoyl-2,3-benzodiazepine; GYKI52466, 1-(4-Aminophenyl)-4-methyl-7,8-methylenedioxy-5H-2,3-benzodiazepine hydrochloride; GYKI53655 (LY300168 hydrochloride), 5-(4-Aminophenyl)-N,8-dimethyl-8,9-dihydro-7H-[1,3]dioxolo[4,5-h][2,3]benzodiazepine-7-carboxamide; LY377770 - (3S,4aR,6S,8aR)-6-(((1H-tetrazol-5-ylmethyl)oxy)methyl)- 1,2,3,4,4a,5,6,7,8,8a-decahydroisoquinoline-3-carboxylic acid; selurampanel (BGG492), N-[7-Isopropyl-6-(2-methylpyrazol-3-yl)-2,4-dioxo-1H-quinazolin-3-yl]methanesulfonamide; GYKI53405 (LY 293606), (7-acetyl-5-(4-aminophenyl)-8-methyl-8,9-dihydro-7H-1,3-dioxolo[4,5-b][2,3]benzodiazepine); CNQX, 6-Cyano-7-nitroquinoxaline-2,3-dione; RPR 119990, 9-carboxymethyl-4-oxo-5H,10H-imidazo[1,2-a]indeno[1,2-e]pyrazin-2-phosphonic acid; FN1040, 29-fluoro (29-F) modified RNA aptamers; FN58, 29-fluoro (29-F) modified RNA aptamers; ACET, (S)-1-(2-Amino-2-carboxyethyl)-3-(2 -carboxy-5-phenylthiophene-3-yl-methyl)-5-methylpyrimidine-2,4-dione; ADE, adverse drug events; 1st AP, first action potential; MES, maximal electroshock seizures; PTZ, Pentylenetetrazol Induced Seizure; SN, substantia nigra.

In the PD model of mice lacking 6-hydroxy dopamine (6-OHDA), treated with neurotoxin 6-hydroxydopamine (6-OHDA), which causes the destruction of nigrostriatal dopaminergic neurons (Simola et al., 2007), it was shown that the L-DOPA treatment caused hyperactivity of AMPA receptors. This hyperactivity was possibly due to the alternative splicing of GluA2 or serine phosphorylation of GluA1, which are known to induce a broad range of changes in the AMPA receptor function (Kobylecki et al., 2010; Silverdale et al., 2010). These findings reinforce the role of Ca^2+^-permeable AMPA receptors in LID and emphasize their potential to serve as therapeutic targets in treating PD-related dyskinesia.

**FIGURE 3:**
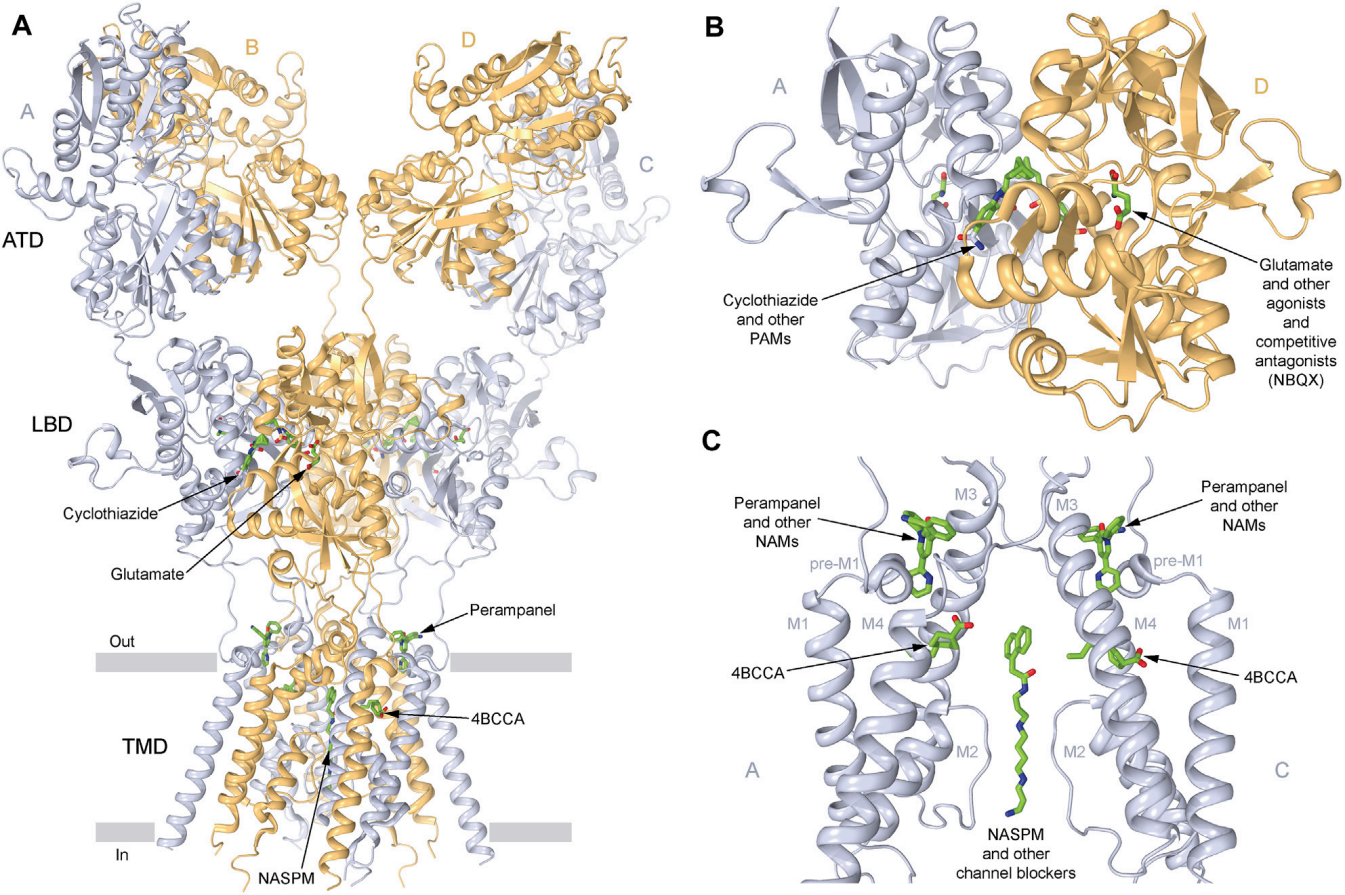
Sites of AMPA receptor pharmacological regulation. **(A)** Structure of the GluA2 AMPA receptor (PDB ID: 6DM1) in ribbon representation, viewed parallel to the membrane, with A and C subunits represented in light blue, and B and D subunits represented in beige, and the layers of the amino-terminal domain (ATD), the ligand-binding domain (LBD), and the transmembrane domain (TMD) labeled. Small-molecule regulators are shown in sticks (green). **(B)** Expanded view of the LBD dimer, with the LBD clamshell binding site of agonists like glutamate and competitive antagonists like NBQX, and the LBD interface binding site of positive allosteric modulators (PAMs) like cyclothiazide (CTZ) being indicated. **(C)** Expanded view of the TMD, with the binding sites of negative allosteric modulators (NAMs) perampanel (PMP; PDB ID: 5L1F) and trans-4-butylcyclohexane carboxylic acid (4BCCA, PDB ID: 6XSR), as well as ion channel blockers like 1-naphthyl acetyl spermine (NASPM; PDB ID: 6DM1) being indicated. Only two subunits (A,C) are shown, with the front and back subunits (B,D) removed for clarity.

Despite extensive efforts in the development of AMPA receptor antagonists, they alone have not been shown effective in the animal models of PD. For instance, while the high-affinity AMPA receptor antagonist quinoxalinedione NBQX (Figure 3) was found to protect neurons from damage (Ossowska, 1994; Jayakar and Dikshit, 2004), low solubility at physiological pH combined with fast renal excretion caused its crystallization in the kidneys at therapeutic doses (Klockgether et al., 1991; Stauch Slusher et al., 1995; Catarzi et al., 2007). However, when NBQX was used in combination with the inhibitors of dopamine and γ-aminobutyric acid (GABA) receptors, the dyskinesia symptoms improved. Indeed, the synergistic effects of buprenorphine hydrochloride (Hospira), 6-OHDA hydrobromide, and methyl ester L-DOPA hydrochloride confirmed the involvement of dopamine, GABA, and glutamate in the development of dyskinesia (Lindenbach et al., 2016), highlighting the complexity of this multisystem disorder. Similarly, the application of the channel blocker 1-naphthyl acetyl spermine trihydrochloride (NASPM), a synthetic analog of Joro spider toxin (Figure 3), which selectively blocks the Ca^2+^-permeable AMPA/kainate receptors, to the *lateral habenula* region had an antidepressant effect in mice with injured *substantia nigra* (Zhang et al., 2019b). This antidepressant effect was also accompanied with an increase in concentrations of dopamine and serotonin in the medial prefrontal cortex. It appears that future development of more potent and more soluble AMPA receptor blockers shows potential to create an effective treatment of PD. This is in stark contrast to AMPA receptor antagonists, which, at high therapeutic doses along with the positive effect on dyskinesia, also produce CNS depression with negative effects on neuronal plasticity.

Positive allosteric modulators (PAMs), including nootropic pyrrolidone compounds like aniracetam, oxiracetam, and piracetam, and benzothiazoles like cyclothiazide and diazoxide (Figure 3), which slow deactivation and reduce desensitization of both Ca^2+^-permeable and Ca^2+^-impermeable AMPA receptors, are known to have neuroprotective and neurotrophic effects, helping in disorders characterized by a decline in cognitive functions, such as PD (Partin, 2015). PD is defined as a movement disorder, but it is also characterized by a variety non-motor symptoms (NMS) in virtually all patients, including hyposmia, constipation, urinary dysfunction, orthostatic hypotension, memory loss, depression, pain, and sleep disturbances (Tolosa et al., 2021). Several AMPA receptor PAMs are known to improve neuronal plasticity. Biarylpropyl sulfonamide ligands, namely, LY404187 and LY503430, protected the *substantia nigra* from damage and strengthened synaptic transmission. LY503430 demonstrated a neuroprotective effect in mice with PD (Murray et al., 2003). Low concentrations of this compound selectively increased the glutamate-dependent flow of calcium ions into the cells via subunits GluA1, GluA3, or GluA4, containing AMPA receptors. The neurotrophic effect of LY503430 was in part due to the stimulation of neurotrophic factors BDNF and GAP-4316 production (Zhang et al., 2019a; O’Neill and Witkin, 2007; O’Neill et al., 2005; Murray et al., 2003). The disadvantage of PAMs is that only a small number of them can penetrate the blood–brain barrier.

Alternative pharmacological agents include phytocannabidiol (CBD), an active compound of the *Cannabis sativa* plant (marijuana), which showed neuroprotective effects in mouse models of several neurodegenerative diseases, including PD (Hampson et al., 1998; Hampson et al., 2000; Lastres-Becker et al., 2005; Garcia et al., 2011; Ruiz-Valdepenas et al., 2011; Pacher et al., 2020). The beneficial effects of CBD observed in the preclinical models of multiple sclerosis (Elliott et al., 2018), PD, and AD can be attributed to the attenuated oxidative/nitrative stress, excitotoxicity, and microglial activation. CBD also significantly reduced AMPA receptor-mediated excitatory postsynaptic currents (EPSCs) and the amplitude and frequency of miniature EPSCs (mEPSCs) in hippocampal neurons, likely affecting the progression of neurodegenerative disorders. Treatment with CBD did not improve motor function or general symptoms in the clinical studies of PD patients but showed improvement in their quality of life and sleep, likely due to a psychotic effect (Peres et al., 2018). Furthermore, CBD was shown to inhibit currents through recombinant GluA1 receptors with an *IC*_50_ value of 22.5 µM and significantly accelerated the deactivation of AMPA receptors composed of GluA1 and GluA2 subunits (Patra et al., 2019). Interestingly, CBD slowed recovery from desensitization for Ca^2+^-permeable GluA1 but not Ca^2+^-impermeable GluA2 receptors. These effects of CBD on receptor kinetics were even more prominent when AMPA receptors were coexpressed with the auxiliary subunit TARP γ8, which is highly expressed in the hippocampus. It is known that hippocampal damage is a common feature among neurodegenerative dementias (Moodley and Chan, 2014). The inhibitory effect on AMPA receptors depended on the CBD interaction with the N-terminal domain (NTD) of GluA1/GluA2 and was completely eliminated by NTD deletion (Yu et al., 2020).

Another compound found in the *C. sativa* plant, Tetrahydrocannabinol (THC), has been shown to reduce NMDA, AMPA, and kainate receptor-mediated neurotoxicity (Hampson et al., 2000). An endogenous cannabinoid receptor agonist with similar pharmacological effects as THC is anandamide (AEA, the major psychoactive component of marijuana) that directly inhibits currents through homomeric GluA1 and GluA3 receptors at rather high concentrations, with IC_50_ values of 161 and 143 μM, respectively, and heteromeric GluA1/3 and GluA2/3 receptors, with the similar IC_50_ values of 148 and 241 μM, respectively (Akinshola et al., 1999a; Akinshola et al., 1999b). One limitation to using AEA is that it also activates TRPV1 channels, which are highly permeable to Ca^2+^ and can contribute to neuronal Ca^2+^ overload (Naziroglu, 2015). Another interesting example is the phytocannabinoid delta 9-tetrahydrocannabivarin (D9-THCV), which undergoes testing in preclinical models of PD (Garcia et al., 2011). There could also be clinical advantages in administering D9-THCV together with CBD as this might lead to symptomatic relief (due to D9-THCV blockade of CB1) and neuroprotection (due to the antioxidant and anti-inflammatory properties of both CBD and D9-THCV). The main difficulty in assessing the drug efficiency is the different time scales of animal and clinical studies. In all animal studies, the effect of drug is monitored shortly after manipulations that induce PD-like symptoms, while in clinical practices, PD is diagnosed 10 years after neurodegeneration has started. Currently, CBD can be considered a preventive agent, without a definitive target, as it affects many enzymes and ion channels, including iGluRs (Peres et al., 2018).

Kainate receptors participate in the regulation of dopaminergic neuron firing frequency, and the expression of the GluK2 subunit is increased in parkinQ311X mouse (a PD model of human parkin-induced toxicity) (Maraschi et al., 2014). Accumulation of GluK2 in the plasma membrane of PD neurons has been shown to be due to slowed GluK2 turnover caused by the loss of parkin protein (ubiquitin E3 ligase that breaks down unnecessary proteins by tagging the damaged and excess proteins with a molecule called ubiquitin), which interacts with the C-terminal tail of GluK2 and is able to ubiquitinate it (Maraschi et al., 2014). Thus, the chronic administration of the kainate receptor antagonist, UBP310, prevented the loss of dopaminergic neurons and increased the survival of the total neuron population in the *substantia nigra* in the acute MPTP mouse model of PD (Stayte et al., 2020). UBP310 is a 4,000-fold more potent antagonist at kainate *vs.* AMPA receptors and is ineffective at NMDA and metabotropic glutamate receptors (Dolman et al., 2007; Regoni et al., 2020), making kainate receptors a perspective novel target for neuroprotective therapy.

Microglia and astrocytes were also shown to contribute to neuroinflammation, which can be beneficial short-term by promoting tissue repair and becomes detrimental when sustained (Kwon and Koh, 2020). Various anti-inflammatory treatments, such as using dexamethasone, ibuprofen, amantadine, minocycline, pituitary adenylate cyclase-activating peptide, and vasoactive intestinal peptide, have been shown to prevent the dopaminergic neuron cell death in animal models. Astrocytes also protect motor neurons from excitotoxic damage via the release of an unidentified soluble factor(s) that induces motor neurons to upregulate the glutamate receptor subunit GluA2 (Rosenblum and Trotti, 2017). The incorporation of GluA2 subunits into glutamate receptors reduces their calcium permeability, providing protection from excitotoxicity by decreasing the influx of calcium into neurons.

In summary, the modulation of AMPA and kainate receptors (when thoroughly tuned to achieve specific goals) appears to be an effective strategy in both inhibiting the progression of PD and restoring CNS degeneration (O'Neill et al., 2004).

## 3 Epilepsy

Epilepsy is a chronic brain disorder characterized by the recurrence of unprovoked seizures caused by abnormal, highly synchronized firing of neurons within a restricted brain region, brain hemisphere, or generalized to the entire brain (Moshe et al., 2015). Seizures occur when clusters of neurons transmit irregular signals. During seizure, many neurons fire (signal) simultaneously at a faster than normal rate, as many as 500 times per second. This surge of excessive and synchronized electrical activity results in involuntary movements, spasms, sensations, emotions, and behaviors, and may cause a loss of consciousness. The word “epilepsy” comes from a Greek word meaning “to seize” or “to attack.” Ancient Greeks believed that the origin of epilepsy is the brain, and it is a divine contribution and a sign of ingenuity. There are several types of epilepsy, and the onset of seizures can be a result of different factors, including prior illnesses, brain injury, abnormal brain development, and, more specifically, autoimmune attack on glutamate receptors (Alexopoulos et al., 2011; Lancaster et al., 2011). However, in many cases, the causes of epilepsy are unknown. For many patients with epilepsy, seizures can be controlled by monotherapy at optimized dosages. Antiepileptic drugs restrain the neuronal activity through various mechanisms, including block of sodium channels or TRPV1, inhibition of excitatory neurotransmission (mainly glutamatergic), or facilitating inhibitory neurotransmission, specifically GABAergic or activated by gamma aminobutyric acid. Their clinical use, however, is limited by side effects. In addition, approximately one-third of patients with refractory epilepsies and other complicated cases, which do not respond to monotherapy, remain untreated (Asth et al., 2021). Therefore, alternative treatment strategies are urgently needed.

Microdialysis and magnetic resonance spectroscopy (MRS) studies showed increased levels of extracellular glutamate in patients with epilepsy (Sarlo and Holton, 2021). Kindling model, an experimental animal model for partial epilepsies, showed both a decrease in GABAergic inhibition and an increase in glutamatergic excitation. These processes are thought to be critically involved in cellular mechanisms underlying the initiation (epileptogenesis) and spread of epileptic seizures that lead to chronic epilepsy (Rogawski et al., 1995). GABA is the major inhibitory neurotransmitter in the brain, which works in balance with the major excitatory neurotransmitter glutamate in healthy individuals. In pharmacological experiments, agonists of iGluRs were shown to be involved in the initiation of seizures and their propagation (Loscher et al., 1999). For example, the infusion of AMPA, kainate, (RS)-2-amino-3-(3-hydroxy-5-tert-butylisoxazol-4-yl) propanoic acid (ATPA), or NMDA elicited involuntary muscle contractions (clonus) and passive partial muscle contractions (tonus) in rodents. Kainate, which acts as a non-desensitizing agonist of AMPA receptors, has been widely used in animal models to induce epilepsy (Kandratavicius et al., 2014). The marine toxin domoic acid, a kainic acid analog from algae or algae-eating fishes, when ingested caused intoxication in humans. Intoxicated patients experienced drug-resistant status epilepticus and developed temporal lobe epilepsy within one year. The consequences of domoic acid intoxication in humans are, therefore, very similar to the kainate-induced status epilepticus in rodents (Ramsdell and Gulland, 2014). These results suggested that the overactivation of AMPA receptors can elicit temporal lobe epilepsy, which is also consistent with a relatively dense expression of AMPA receptors in the hippocampus. There is preclinical and clinical evidence that AMPA receptor antagonists inhibit seizures (Rogawski, 2013; Barker-Haliski and White, 2015). One such AMPA receptor antagonists, perampanel, was approved for the treatment of different forms of epilepsy (Bialer et al., 2010; Hibi et al., 2012; Chen et al., 2014). Perampanel (Figure 3) is a negative allosteric modulator (NAM) that inhibits AMPA receptors with high selectivity by stabilizing their closed state and thereby preventing the opening of the ion channel (Yelshanskaya et al., 2016). It also blocks kainate receptors, but with lower affinity (Yelshanskaya et al., 2016; Taniguchi et al., 2022; Gangwar et al., 2023), and shows little selectivity when acting on different AMPA receptor subtypes (Hanada et al., 2014). In 2012, perampanel was approved as an adjunctive treatment of partial-onset seizures and, in 2015, as a treatment of the primary generalized tonic-clonic seizures in patients 12 years and older (Greenwood and Valdes, 2016). Investigations on the use of perampanel for treatment of other types of seizure disorders are ongoing (Potschka and Trinka, 2019). The antiseizure efficacy of perampanel is dose-dependent, and at high doses, this drug can cause side effects like dizziness, somnolence, headache, fatigue, nausea, and vertigo (Greenwood and Valdes, 2016). The competitive antagonist BGG492 (selurampanel) was advanced into clinical trials by Novartis in 2015 for the treatment of epilepsy. It has also been studied as an acute treatment of migraine and is found to produce some pain relief but with a relatively high rate of side effects. The most common adverse effects are disorders of the nervous system (dizziness, mostly mild to moderate in severity) and gastrointestinal tract (Faught, 2014; Gomez-Mancilla et al., 2014). Similar to quinoxalinediones, BGG492 inhibits both AMPA and kainate receptors.

Dietary strategies can provide seizure control in patients who do not respond to antiseizure drugs (Sills et al., 1986; Martin-McGill et al., 2020; Leitner et al., 2023). There are several types of dietary therapies, all of which are high in fat, to some extent restricted in carbohydrates, and associated with ketosis. Medium-chain triglycerides (MCTs), which are highly abundant in ketogenic diet, including decanoic acid, have long been known to have an acute anticonvulsant effect in animal models (Chang et al., 2016). Interestingly, in the Indian medical system of Ayurveda, epilepsy was treated with ghee, which is about 50% composed of saturated fat (palmitic and oleic acids). Chang et al. (2016) demonstrated that direct inhibition of excitatory neurotransmission by decanoic acid at therapeutically relevant concentrations is a result of non-competitive antagonism of AMPA receptors that likely underlies the antiseizure effects. This inhibitory effect was hypothesized to occur via binding of decanoic acid to sites on the transmembrane M3 helix of the GluA2 AMPA receptor transmembrane domain, distinct from the binding sites of perampanel (Chang et al., 2016). The synergistic effects of perampanel and decanoic acid inhibition of AMPA receptors through different binding sites were demonstrated in an *ex vivo* model of seizure activity and by inhibiting seizure-induced activity in the human brain slices (Augustin et al., 2018), and may represent a prospective antiepileptic strategy. A novel family of fatty acids, branched derivatives of octanoic acid (OA) related to the MCT ketogenic diet, was also identified as a possible treatment of epilepsy (Chang et al., 2015). An OA derivative with the strongest antiepileptic effects, trans 4-butylcyclohexane carboxylic acid (4-BCCA), was shown to inhibit AMPA receptors with low affinity, acting via transmembrane domain binding sites, distinct from perampanel and ion channel blockers (Figure 3) (Yelshanskaya et al., 2022).

CBD, an active compound of marijuana, significantly prolonged the seizure latency and reduced the severity of thermally induced seizures in a mouse hyperthermia-induced seizure model (Patra et al., 2019), partly due to its effect on AMPA receptors (description in the PD section). CBD has been recently approved in the United States and the European Union as an add-on antiepileptic drug (epidiolex) for the treatment of patients affected by refractory epilepsy, such as Dravet and Lennox–Gastaut syndrome (Pagano et al., 2022), and caused resurgence of interest in pharmacology of cannabinoids in general and phytocannabinoids in particular.

Kainate receptors represent another key class of glutamate receptors that may play an important role in the pathophysiology of epilepsy. It has been shown that these receptors, especially GluK4, are upregulated in the astrocytes of the hippocampus and surrounding cortex during status epilepticus (SE), associated with seizures that last more than 5 min and occur with high frequency (Gibbons et al., 2013; Vargas et al., 2013). Although the functional role of kainate receptors in seizures remains to be determined, selective targeting of astrocytic processes that contribute to glutamate release represents a novel therapeutic strategy for the treatment of epilepsy (Gibbons et al., 2013). More recently, several studies suggested that the presynaptic kainate receptors work cooperatively with the cannabinoid receptors to control the release of glutamate (Marshall et al., 2018) and GABA (Daw et al., 2010; Lourenco et al., 2010; Lourenco et al., 2011; Wyeth et al., 2017).

There is evidence for the direct link between AMPA receptor mutations (GluA2 subtype) and epilepsy, although the corresponding studies are limited, with the majority of identified AMPA receptor mutations linked to cognitive impairment and autism (Salpietro et al., 2019). Genetic changes in the GluA2 subunit were mapped to different parts of the receptor, transmembrane, ligand-binding, and N-terminal domains, suggesting that they produce different effects on the AMPA receptor function, including changes in trafficking (e.g., by increasing the surface expression), rigidifying the receptor’s ligand-binding domains, or altering affinity to glutamate or natural regulators, thus influencing synaptic plasticity.

One notable event that follows seizures in humans and in mouse models of epilepsy is the dramatic increase in expression of the GluA1 flip isoforms. These isoforms not only confer greater glutamate sensitivity than the flop isoforms but, if present in excess, tend to form homomeric Ca^2+^-permeable AMPA receptors. Either of these features can enhance the excitatory synaptic currents. It has been reported that a splice-modulating oligonucleotide decreased the GluA1 expression and showed antiseizure effects, including reduced postseizure hyperexcitability in neonatal mice (Lykens et al., 2017). Such targeting of specific AMPA subunit isoforms may have a potential to alter the expression of AMPA receptor subtypes involved in the disease states. Likewise, various molecular approaches, including the use of small interfering peptides (Fosgerau and Hoffmann, 2015), have been used successfully to target protein–protein interactions and prevent the endocytosis of AMPA receptors involved in behavioral sensitization models of drug addiction. Small interfering peptides (GluR2-3Y) have also been developed to selectively prevent the endocytosis of AMPA receptors containing GluA2 subunits (Lin et al., 2016). An exciting future possibility is to further develop such approaches and to target specific auxiliary subunits that may be involved in the delivery of Ca^2+^-permeable AMPA receptors.

## 4 Amyotrophic lateral sclerosis

ALS, also known as Lou Gehrig’s Disease, is a progressive and fatal neurodegenerative disease, which predominantly affects motor neurons that control voluntary muscle movement, including those located in the spinal cord, brain stem, and motor cortex. Different clinical symptoms of ALS depend on whether the upper or lower motor neurons are damaged (Grad et al., 2017). Neuron injury leads to muscle weakness, progressive paralysis, respiratory failure, and death within 3–5 years after the disease onset (Kawahara and Kwak, 2005). To date, the exact mechanisms of ALS pathogenesis remain unknown. Early biochemical studies revealed increased glutamate levels in the ALS patient’s cerebrospinal fluid (Rothstein et al., 1990; Shaw et al., 1995). The dysfunction of RNA and protein homeostasis, which results in glutamate-mediated excitotoxicity, alongside protein aggregation, mitochondrial dysfunction, and oxidative stress, is also responsible for the ALS-specific neurodegeneration (van den Bos et al., 2019; Zhang and Abdullah, 2013; Taylor et al., 2016). About 10% of ALS cases are inherited within families, almost always as dominant traits and frequently with high penetrance.

The first ALS-associated gene, coding for the cytosolic superoxide dismutase (SOD1), was reported in 1993, with more related genes discovered since (Rosen et al., 1993). SOD1 encodes the ubiquitously expressed cytoplasmic superoxide dismutase, which represents a major cell antioxidant. When misfolded due to mutations, SOD1 leads to the toxic accumulation of aggregated protein, which is a possible toxic contributor to ALS (Taylor et al., 2016). Large protein aggregates in neuronal cells are a hallmark in many neurodegenerative diseases, including PD and AD. However, recent studies showed that the disease-causing mutants of SOD1 are not sufficient to drive the disease acting in motor neurons only and have to act in their glial partners, oligodendrocytes, and astrocytes as well (Yamanaka et al., 2008; Wang et al., 2011). The latter cells represent the final layer of the blood–brain barrier, which supplies nutrients to neurons, buffering ions, and recycling the neurotransmitter glutamate. Astrocytes limit neuronal firing by the rapid recovery of synaptic glutamate and release of factors upregulating GluA2 subunit expression. It was shown that astrocytes from familiar and sporadic ALS patients are toxic to cocultured healthy motor neurons (Haidet-Phillips et al., 2011; Re et al., 2014). Motor neurons seem to exhibit a particular sensitivity to excitotoxicity: they are large in size (long axons) and have high energy requirements, relatively low Ca^2+^-buffering capacity, and contain molecular chaperones with mitochondrial activity and neurofilament content involved in excitotoxic sensitivity (Menon et al., 2014).

Riluzole, the only drug to prolong, although modestly, the survival of ALS patients, is a potent neuroprotective agent with multimodal effects on neuronal activity. One of the mechanisms of riluzole action, acceleration of glutamate clearance and prevention of excessive excitatory neurotransmitter release from presynaptic terminals, causes an effective reduction in the rate of disease progression. Riluzole was also shown to interact with voltage-dependent sodium channels, highlighting its non-specific action on ligand-gated ion channels (Menon et al., 2014; Dharmadasa and Kiernan, 2018; Fang et al., 2018; Lazarevic et al., 2018; Tarantino et al., 2022).

With a higher sensitivity to excitotoxicity, spinal motor neurons exhibit lower levels of GluA2 subunit expression and consequently higher levels of Ca^2+^-permeable AMPA receptors than most neuronal subgroups (Kawahara and Kwak, 2005). It was suggested that one possible mechanism of ALS progression is an increased number of Ca^2+^-permeable AMPA receptors due to an abnormal increase in GluA1 and decrease in GluA2 subunit expressions (Kawahara and Kwak, 2005). In cases of sporadic ALS, there are reduced levels of adenosine deaminase acting on RNA type 2 enzyme (ADAR2) expression, which disrupts the efficient Q/R editing of GluA2 pre-mRNA, also causing increased Ca^2+^ permeability. The downregulation of ADAR2 is believed to be caused by the transactive response DNA-binding protein TDP-43, a transcriptional regulator, and transport protein that plays an important role in alternative splicing. The behavior of this protein is the most reliable hallmark of the motor neuron pathology during ALS, characterized by abnormally insoluble, mislocalized, hyperphosphorylated, or fragmented TDP-43 (Neumann et al., 2006; Kwong et al., 2008; Chen-Plotkin et al., 2010). Notably, the pathological forms of TDP-43 downregulate ADAR2, leading to a failure in Q/R editing of GluA2 pre-mRNA (Yamashita and Kwak, 2019; Guo and Ma, 2021). The unedited GluA2 AMPA receptor subunit is, therefore, the potential target for the ALS drug development. TDP-43 is the critical component of macromolecular complexes that generate small non-coding RNAs (microRNAs) that function in RNA silencing. The loss of TDP-43 results in the reduced expression of microRNAs in model systems.

In addition, mutant cultural astrocytes induce changes in GluA1 and GluA2 AMPA receptor subunit expression on the surface of motor neurons via the secretion of the tumor necrosis factor alpha (TNF-α) in the astrocyte condition medium, thus leading to their increased vulnerability to excitotoxic damage (Kia et al., 2018). Previous research demonstrated that the application of exogenous TNF-α to neurons instigated a rapid insertion of AMPA receptors into the plasma membrane, in some cases specifically GluA2-lacking Ca^2+^-permeable AMPA receptors, thus potentiating glutamate-dependent excitotoxic damage (Yin et al., 2012). These findings suggest that targeting the TNF-α-induced AMPA receptor expression might be a novel direction for the design of neuroprotective drugs (Zhao et al., 2010).

Antiepileptic drugs, such as perampanel, improve the ALS phenotype but also cause sedation (Akamatsu et al., 2016). Another AMPA receptor antagonist, talampanel, which was initially found to be beneficial for the ALS patients in phase II clinical trials, failed in phase III clinical trials due to low efficacy. Talampanel has a much shorter half-life in humans (approximately 3–4 h) than perampanel (approximately 105 h) (Pascuzzi et al., 2010). Based on the experiments in mice, two novel chemically modified RNA aptamers, which are easily-soluble in water, showing high potency and selectivity AMPA antagonists, have been recently introduced as a new treatment of ALS, an alternative to traditional, small-molecule compounds (Akamatsu et al., 2022). Since RNA aptamers do not cross the blood–brain barrier, researchers hypothesized that *in vivo*, the dose of these aptamers can be administered as low as possible to achieve the therapeutic efficacy with minimal or no adverse effects by direct injection into the spinal cord (Akamatsu et al., 2022). Another AMPA/kainate receptor antagonist, NBQX, was evaluated in the mouse model of ALS and found to prevent the kainate-induced motor neuron death and to prolong the animal survival (Van Damme et al., 2003). Clinical trials of other small molecules as potential drugs are ongoing. They include compounds acting on TRP channels, K^+^ channels, Cl^−^ channels, acetylcholine receptors, Na^+^ channels, and metabotropic glutamate receptors (Tarantino et al., 2022).

## 5 Conclusion

Non-NMDA iGluRs, AMPA and kainate receptors, play an important role in the pathogenic mechanisms of epilepsy and neurodegenerative diseases like PD, AD, and ALS. Modulation of their activity, subunit expression, and trafficking using selective antagonists appears to be an effective strategy in alleviating the disease symptoms. However, many of these drugs have negative side effects and/or solubility/bioavailability problems. A reasonable approach to new types of therapeutic interventions targeting the expression and trafficking of non-NMDA receptors, as well as novel types of antagonists (e.g., RNA aptamers) and small molecules that selectively bind and regulate Ca^2+^-permeable AMPA and kainate receptors, will likely uncover alternative strategies to relieve the burden of both acute and chronic neurodegeneration and ultimately lead to neuroprotection.

## References

[B1] Aizawa H. Kato H. Oba K. Kawahara T. Okubo Y. Saito T. (2022). Randomized phase 2 study of perampanel for sporadic amyotrophic lateral sclerosis. J. Neurol. 269, 885–896. 10.1007/s00415-021-10670-y 34191081 PMC8782807

[B2] Akamatsu M. Yamashita T. Hirose N. Teramoto S. Kwak S. (2016). The AMPA receptor antagonist perampanel robustly rescues amyotrophic lateral sclerosis (ALS) pathology in sporadic ALS model mice. Sci. Rep. 6, 28649. 10.1038/srep28649 27350567 PMC4923865

[B3] Akamatsu M. Yamashita T. Teramoto S. Huang Z. Lynch J. Toda T. (2022). Testing of the therapeutic efficacy and safety of AMPA receptor RNA aptamers in an ALS mouse model. Life Sci. Alliance 5 (4), e202101193. 10.26508/lsa.202101193 35022247 PMC8761490

[B4] Akinshola B. E. Chakrabarti A. Onaivi E. S. (1999a). In-vitro and in-vivo action of cannabinoids. Neurochem. Res. 24, 1233–1240. 10.1023/a:1020968922151 10492518

[B5] Akinshola B. E. Taylor R. E. Ogunseitan A. B. Onaivi E. S. (1999b). Anandamide inhibition of recombinant AMPA receptor subunits in Xenopus oocytes is increased by forskolin and 8-bromo-cyclic AMP. Naunyn Schmiedeb. Arch. Pharmacol. 360, 242–248. 10.1007/s002109900078 10543424

[B6] Albo F. Pieri M. Zona C. (2004). Modulation of AMPA receptors in spinal motor neurons by the neuroprotective agent riluzole. J. Neurosci. Res. 78, 200–207. 10.1002/jnr.20244 15378511

[B7] Alexopoulos H. Kosmidis M. L. Dalmau J. Dalakas M. C. (2011). Paraneoplastic anti-NMDAR encephalitis: long term follow-up reveals persistent serum antibodies. J. Neurol. 258, 1568–1570. 10.1007/s00415-011-5982-4 21384161

[B8] Anselmi L. Bove C. Coleman F. H. Le K. Subramanian M. P. Venkiteswaran K. (2018). Ingestion of subthreshold doses of environmental toxins induces ascending Parkinsonism in the rat. NPJ Park. Dis. 4, 30. 10.1038/s41531-018-0066-0 PMC6160447 30302391

[B9] Asth L. Iglesias L. P. De Oliveira A. C. Moraes M. F. D. Moreira F. A. (2021). Exploiting cannabinoid and vanilloid mechanisms for epilepsy treatment. Epilepsy Behav. 121, 106832. 10.1016/j.yebeh.2019.106832 31839498

[B10] Atanasova T. Savonlehto T. Kukko-Lukjanov T. K. Kharybina Z. Chang W. C. Lauri S. E. (2023). Progressive development of synchronous activity in the hippocampal neuronal network is modulated by GluK1 kainate receptors. Neuropharmacology 239, 109671. 10.1016/j.neuropharm.2023.109671 37567438

[B11] Augustin K. Williams S. Cunningham M. Devlin A. M. Friedrich M. Jayasekera A. (2018). Perampanel and decanoic acid show synergistic action against AMPA receptors and seizures. Epilepsia 59, e172–e178. 10.1111/epi.14578 30324610

[B12] Barker-Haliski M. White H. S. (2015). Glutamatergic mechanisms associated with seizures and epilepsy. Cold Spring Harb. Perspect. Med. 5, a022863. 10.1101/cshperspect.a022863 26101204 PMC4526718

[B13] Barton M. E. Peters S. C. Shannon H. E. (2003). Comparison of the effect of glutamate receptor modulators in the 6 Hz and maximal electroshock seizure models. Epilepsy Res. 56, 17–26. 10.1016/j.eplepsyres.2003.08.001 14529950

[B14] Becker L. A. Huang B. Bieri G. Ma R. Knowles D. A. Jafar-Nejad P. (2017). Therapeutic reduction of ataxin-2 extends lifespan and reduces pathology in TDP-43 mice. Nature 544, 367–371. 10.1038/nature22038 28405022 PMC5642042

[B15] Bhunia S. Kolishetti N. Arias A. Y. Vashist A. Nair M. (2022). Cannabidiol for neurodegenerative disorders: a comprehensive review. Front. Pharmacol. 13, 989717. 10.3389/fphar.2022.989717 36386183 PMC9640911

[B16] Bialer M. Johannessen S. I. Kupferberg H. J. Levy R. H. Loiseau P. Perucca E. (2002). Progress report on new antiepileptic drugs: a summary of the Sixth Eilat Conference (EILAT VI). Epilepsy Res. 51, 31–71. 10.1016/s0920-1211(02)00106-7 12350382

[B17] Bialer M. Johannessen S. I. Kupferberg H. J. Levy R. H. Perucca E. Tomson T. (2004). Progress report on new antiepileptic drugs: a summary of the Seventh Eilat Conference (EILAT VII). Epilepsy Res. 61, 1–48. 10.1016/j.eplepsyres.2004.07.010 15570674

[B18] Bialer M. Johannessen S. I. Kupferberg H. J. Levy R. H. Perucca E. Tomson T. (2007). Progress report on new antiepileptic drugs: a summary of the Eigth Eilat Conference (EILAT VIII). Epilepsy Res. 73, 1–52. 10.1016/j.eplepsyres.2006.10.008 17158031

[B19] Bialer M. Johannessen S. I. Levy R. H. Perucca E. Tomson T. White H. S. (2010). Progress report on new antiepileptic drugs: a summary of the Tenth Eilat Conference (EILAT X). Epilepsy Res. 92, 89–124. 10.1016/j.eplepsyres.2010.09.001 20970964

[B20] Bjorklund G. Stejskal V. Urbina M. A. Dadar M. Chirumbolo S. Mutter J. (2018). Metals and Parkinson’s disease: mechanisms and biochemical processes. Curr. Med. Chem. 25, 2198–2214. 10.2174/0929867325666171129124616 29189118

[B21] Blauwendraat C. Nalls M. A. Singleton A. B. (2020). The genetic architecture of Parkinson’s disease. Lancet Neurol. 19, 170–178. 10.1016/S1474-4422(19)30287-X 31521533 PMC8972299

[B22] Buchan A. M. Lesiuk H. Barnes K. A. Li H. Huang Z. G. Smith K. E. (1993). AMPA antagonists: do they hold more promise for clinical stroke trials than NMDA antagonists? Stroke 24, I148–I152. 7504338

[B23] Calabrese F. Savino E. Mocaer E. Bretin S. Racagni G. Riva M. A. (2017). Upregulation of neurotrophins by S 47445, a novel positive allosteric modulator of AMPA receptors in aged rats. Pharmacol. Res. 121, 59–69. 10.1016/j.phrs.2017.04.019 28442348

[B24] Calon F. Rajput A. H. Hornykiewicz O. Bedard P. J. Di Paolo T. (2003). Levodopa-induced motor complications are associated with alterations of glutamate receptors in Parkinson’s disease. Neurobiol. Dis. 14, 404–416. 10.1016/j.nbd.2003.07.003 14678757

[B25] Canton T. Bohme G. A. Boireau A. Bordier F. Mignani S. Jimonet P. (2001). RPR 119990, a novel alpha-amino-3-hydroxy-5-methyl-4-isoxazolepropionic acid antagonist: synthesis, pharmacological properties, and activity in an animal model of amyotrophic lateral sclerosis. J. Pharmacol. Exp. Ther. 299, 314–322. 11561094

[B26] Catarzi D. Colotta V. Varano F. (2007). Competitive AMPA receptor antagonists. Med. Res. Rev. 27, 239–278. 10.1002/med.20084 16892196

[B27] Chang P. Augustin K. Boddum K. Williams S. Sun M. Terschak J. A. (2016). Seizure control by decanoic acid through direct AMPA receptor inhibition. Brain 139, 431–443. 10.1093/brain/awv325 26608744 PMC4805082

[B28] Chang P. Zuckermann A. M. Williams S. Close A. J. Cano-Jaimez M. McEvoy J. P. (2015). Seizure control by derivatives of medium chain fatty acids associated with the ketogenic diet show novel branching-point structure for enhanced potency. J. Pharmacol. Exp. Ther. 352, 43–52. 10.1124/jpet.114.218768 25326131

[B29] Chen C. Y. Matt L. Hell J. W. Rogawski M. A. (2014). Perampanel inhibition of AMPA receptor currents in cultured hippocampal neurons. PLoS One 9, e108021. 10.1371/journal.pone.0108021 25229608 PMC4168215

[B30] Cheng F. Vivacqua G. Yu S. (2011). The role of alpha-synuclein in neurotransmission and synaptic plasticity. J. Chem. Neuroanat. 42, 242–248. 10.1016/j.jchemneu.2010.12.001 21167933

[B31] Chen-Plotkin A. S. Lee V. M. Trojanowski J. Q. (2010). TAR DNA-binding protein 43 in neurodegenerative disease. Nat. Rev. Neurol. 6, 211–220. 10.1038/nrneurol.2010.18 20234357 PMC2892118

[B32] Choi D. W. Koh J. Y. Peters S. (1988). Pharmacology of glutamate neurotoxicity in cortical cell culture: attenuation by NMDA antagonists. J. Neurosci. 8, 185–196. 10.1523/JNEUROSCI.08-01-00185.1988 2892896 PMC6569373

[B33] Couratier P. Hugon J. Sindou P. Vallat J. M. Dumas M. (1993). Cell culture evidence for neuronal degeneration in amyotrophic lateral sclerosis being linked to glutamate AMPA/kainate receptors. Lancet 341, 265–268. 10.1016/0140-6736(93)92615-z 8093916

[B34] Cull-Candy S. G. Farrant M. (2021). Ca(2+) -permeable AMPA receptors and their auxiliary subunits in synaptic plasticity and disease. J. Physiol. 599, 2655–2671. 10.1113/JP279029 33533533 PMC8436767

[B35] Daw M. I. Pelkey K. A. Chittajallu R. Mcbain C. J. (2010). Presynaptic kainate receptor activation preserves asynchronous GABA release despite the reduction in synchronous release from hippocampal cholecystokinin interneurons. J. Neurosci. 30, 11202–11209. 10.1523/JNEUROSCI.6334-09.2010 20720128 PMC2937572

[B36] De Miranda B. R. Goldman S. M. Miller G. W. Greenamyre J. T. Dorsey E. R. (2022). Preventing Parkinson’s disease: an environmental agenda. J. Park. Dis. 12, 45–68. 10.3233/JPD-212922 PMC8842749 34719434

[B37] Dharmadasa T. Kiernan M. C. (2018). Riluzole, disease stage and survival in ALS. Lancet Neurol. 17, 385–386. 10.1016/S1474-4422(18)30091-7 29525493

[B38] Dhir A. Chavda V. (2016). Pre- and post-exposure talampanel (GYKI 53773) against kainic acid seizures in neonatal rats. Pharmacol. Rep. 68, 190–195. 10.1016/j.pharep.2015.08.011 26721372

[B39] Dolman N. P. More J. C. Alt A. Knauss J. L. Pentikainen O. T. Glasser C. R. (2007). Synthesis and pharmacological characterization of N3-substituted willardiine derivatives: role of the substituent at the 5-position of the uracil ring in the development of highly potent and selective GLUK5 kainate receptor antagonists. J. Med. Chem. 50, 1558–1570. 10.1021/jm061041u 17348638

[B40] Donevan S. D. Rogawski M. A. (1993). GYKI 52466, a 2,3-benzodiazepine, is a highly selective, noncompetitive antagonist of AMPA/kainate receptor responses. Neuron 10, 51–59. 10.1016/0896-6273(93)90241-i 7678966

[B41] Dorsey E. R. Bloem B. R. (2018). The Parkinson pandemic-A call to action. JAMA Neurol. 75, 9–10. 10.1001/jamaneurol.2017.3299 29131880

[B42] Dorsey E. R. Constantinescu R. Thompson J. P. Biglan K. M. Holloway R. G. Kieburtz K. (2007). Projected number of people with Parkinson disease in the most populous nations, 2005 through 2030. Neurology 68, 384–386. 10.1212/01.wnl.0000247740.47667.03 17082464

[B43] Dorsey E. R. Sherer T. Okun M. S. Bloem B. R. (2018). The emerging evidence of the Parkinson pandemic. J. Park. Dis. 8, S3–S8. 10.3233/JPD-181474 PMC6311367 30584159

[B44] Duty S. (2012). Targeting glutamate receptors to tackle the pathogenesis, clinical symptoms and levodopa-induced dyskinesia associated with Parkinson’s disease. CNS Drugs 26, 1017–1032. 10.1007/s40263-012-0016-z 23114872

[B45] Eggert K. Squillacote D. Barone P. Dodel R. Katzenschlager R. Emre M. German Competence Network on Parkinson's Disease. (2010). Safety and efficacy of perampanel in advanced Parkinson's disease: a randomized, placebo-controlled study. Mov. Disord. 25, 896–905. 10.1002/mds.22974 20461807

[B46] Elbaz A. Carcaillon L. Kab S. Moisan F. (2016). Epidemiology of Parkinson’s disease. Rev. Neurol. Paris. 172, 14–26. 10.1016/j.neurol.2015.09.012 26718594

[B47] Elger C. E. Hong S. B. Brandt C. Mancione L. Han J. Strohmaier C. (2017). BGG492 as an adjunctive treatment in patients with partial-onset seizures: a 12-week, randomized, double-blind, placebo-controlled, phase II dose-titration study with an open-label extension. Epilepsia 58, 1217–1226. 10.1111/epi.13771 28500678

[B48] Elliott D. M. Singh N. Nagarkatti M. Nagarkatti P. S. (2018). Cannabidiol attenuates experimental autoimmune encephalomyelitis model of multiple sclerosis through induction of myeloid-derived suppressor cells. Front. Immunol. 9, 1782. 10.3389/fimmu.2018.01782 30123217 PMC6085417

[B49] Fan B. Jabeen R. Bo B. Guo C. Han M. Zhang H. (2020). What and how can physical activity prevention function on Parkinson’s disease? Oxid. Med. Cell Longev. 2020, 4293071. 10.1155/2020/4293071 32215173 PMC7042542

[B50] Fang T. Al Khleifat A. Meurgey J. H. Jones A. Leigh P. N. Bensimon G. (2018). Stage at which riluzole treatment prolongs survival in patients with amyotrophic lateral sclerosis: a retrospective analysis of data from a dose-ranging study. Lancet Neurol. 17, 416–422. 10.1016/S1474-4422(18)30054-1 29525492 PMC5899963

[B51] Faught E. (2014). BGG492 (selurampanel), an AMPA/kainate receptor antagonist drug for epilepsy. Expert Opin. Investig. Drugs 23, 107–113. 10.1517/13543784.2014.848854 24147649

[B52] Fernandez-Ruiz J. Sagredo O. Pazos M. R. Garcia C. Pertwee R. Mechoulam R. (2013). Cannabidiol for neurodegenerative disorders: important new clinical applications for this phytocannabinoid? Br. J. Clin. Pharmacol. 75, 323–333. 10.1111/j.1365-2125.2012.04341.x 22625422 PMC3579248

[B53] Fosgerau K. Hoffmann T. (2015). Peptide therapeutics: current status and future directions. Drug Discov. Today 20, 122–128. 10.1016/j.drudis.2014.10.003 25450771

[B54] Gangwar S. P. Yen L. Y. Yelshanskaya M. V. Sobolevsky A. I. (2023). Positive and negative allosteric modulation of GluK2 kainate receptors by BPAM344 and antiepileptic perampanel. Cell Rep. 42, 112124. 10.1016/j.celrep.2023.112124 36857176 PMC10440371

[B55] Garcia C. Palomo-Garo C. Garcia-Arencibia M. Ramos J. Pertwee R. Fernandez-Ruiz J. (2011). Symptom‐relieving and neuroprotective effects of the phytocannabinoid Δ^9^‐THCV in animal models of Parkinson's disease. Br. J. Pharmacol. 163, 1495–1506. 10.1111/j.1476-5381.2011.01278.x 21323909 PMC3165958

[B56] Gardinier K. M. Gernert D. L. Porter W. J. Reel J. K. Ornstein P. L. Spinazze P. (2016). Discovery of the first alpha-Amino-3-hydroxy-5-methyl-4-isoxazolepropionic acid (AMPA) receptor antagonist dependent upon transmembrane AMPA receptor regulatory protein (TARP) gamma-8. J. Med. Chem. 59, 4753–4768. 10.1021/acs.jmedchem.6b00125 27067148

[B57] Gibbons M. B. Smeal R. M. Takahashi D. K. Vargas J. R. Wilcox K. S. (2013). Contributions of astrocytes to epileptogenesis following status epilepticus: opportunities for preventive therapy? Neurochem. Int. 63, 660–669. 10.1016/j.neuint.2012.12.008 23266599 PMC4353644

[B58] Golbe L. I. (1991). Young-onset Parkinson’s disease: a clinical review. Neurology 41, 168–173. 10.1212/wnl.41.2_part_1.168 1992358

[B59] Gomez-Mancilla B. Brand R. Jurgens T. P. Gobel H. Sommer C. Straube A. (2014). Randomized, multicenter trial to assess the efficacy, safety and tolerability of a single dose of a novel AMPA receptor antagonist BGG492 for the treatment of acute migraine attacks. Cephalalgia 34, 103–113. 10.1177/0333102413499648 23963355

[B60] Grad L. I. Rouleau G. A. Ravits J. Cashman N. R. (2017). Clinical spectrum of amyotrophic lateral sclerosis (ALS). Cold Spring Harb. Perspect. Med. 7, a024117. 10.1101/cshperspect.a024117 28003278 PMC5538408

[B61] Greenwood J. Valdes J. (2016). Perampanel (fycompa): a review of clinical efficacy and safety in epilepsy. P T 41, 683–698. 27904300 PMC5083075

[B62] Guo C. Ma Y. Y. (2021). Calcium permeable-AMPA receptors and excitotoxicity in neurological disorders. Front. Neural Circuits 15, 711564. 10.3389/fncir.2021.711564 34483848 PMC8416103

[B63] Haidet-Phillips A. M. Hester M. E. Miranda C. J. Meyer K. Braun L. Frakes A. (2011). Astrocytes from familial and sporadic ALS patients are toxic to motor neurons. Nat. Biotechnol. 29, 824–828. 10.1038/nbt.1957 21832997 PMC3170425

[B64] Hampson A. J. Grimaldi M. Axelrod J. Wink D. (1998). Cannabidiol and (−)Δ ^9^ -tetrahydrocannabinol are neuroprotective antioxidants. Proc. Natl. Acad. Sci. U.S. A. 95, 8268–8273. 10.1073/pnas.95.14.8268 9653176 PMC20965

[B65] Hampson A. J. Grimaldi M. Lolic M. Wink D. Rosenthal R. Axelrod J. (2000). Neuroprotective antioxidants from marijuana. Ann. N. Y. Acad. Sci. 899, 274–282. 10.1111/j.1749-6632.2000.tb06193.x 10863546

[B66] Hanada T. Hashizume Y. Tokuhara N. Takenaka O. Kohmura N. Ogasawara A. (2011). Perampanel: a novel, orally active, noncompetitive AMPA-receptor antagonist that reduces seizure activity in rodent models of epilepsy. Epilepsia 52, 1331–1340. 10.1111/j.1528-1167.2011.03109.x 21635236

[B67] Hanada T. Ido K. Kosasa T. (2014). Effect of perampanel, a novel AMPA antagonist, on benzodiazepine-resistant status epilepticus in a lithium-pilocarpine rat model. Pharmacol. Res. Perspect. 2, e00063. 10.1002/prp2.63 25505607 PMC4186423

[B68] Hansen K. B. Wollmuth L. P. Bowie D. Furukawa H. Menniti F. S. Sobolevsky A. I. (2021). Structure, function, and pharmacology of glutamate receptor ion channels. Pharmacol. Rev. 73, 1469–1658. 10.1124/pharmrev.120.000131 PMC8626789 34753794

[B69] Hibi S. Ueno K. Nagato S. Kawano K. Ito K. Norimine Y. (2012). Discovery of 2-(2-oxo-1-phenyl-5-pyridin-2-yl-1,2-dihydropyridin-3-yl)benzonitrile (perampanel): a novel, noncompetitive alpha-amino-3-hydroxy-5-methyl-4-isoxazolepropanoic acid (AMPA) receptor antagonist. J. Med. Chem. 55, 10584–10600. 10.1021/jm301268u 23181587

[B70] Hotait M. Ismail H. H. Saab G. E. Salameh J. S. (2021). An open label pilot study of the safety and tolerability of perampanel in amyotrophic lateral sclerosis. Muscle Nerve 64, 504–508. 10.1002/mus.27385 34322897

[B71] Hoye A. T. Davoren J. E. Wipf P. Fink M. P. Kagan V. E. (2008). Targeting mitochondria. Acc. Chem. Res. 41, 87–97. 10.1021/ar700135m 18193822

[B72] Hughes A. J. Ben-Shlomo Y. Daniel S. E. Lees A. J. (1992). What features improve the accuracy of clinical diagnosis in Parkinson’s disease: a clinicopathologic study. Neurology 42, 1142–1146. 10.1212/wnl.42.6.1142 1603339

[B73] Hurley M. J. Brandon B. Gentleman S. M. Dexter D. T. (2013). Parkinson’s disease is associated with altered expression of CaV1 channels and calcium-binding proteins. Brain 136, 2077–2097. 10.1093/brain/awt134 23771339

[B74] Hurwitz B. (2014). Urban observation and sentiment in James Parkinson’s essay on the shaking Palsy (1817). Lit. Med. 32, 74–104. 10.1353/lm.2014.0002 25055707 PMC4077538

[B75] Jakus R. Graf M. Ando R. D. Balogh B. Gacsalyi I. Levay G. (2004). Effect of two noncompetitive AMPA receptor antagonists GYKI 52466 and GYKI 53405 on vigilance, behavior and spike-wave discharges in a genetic rat model of absence epilepsy. Brain Res. 1008, 236–244. 10.1016/j.brainres.2004.01.087 15145761

[B76] Jankovic J. (2008). Parkinson’s disease: clinical features and diagnosis. J. Neurol. Neurosurg. Psychiatry 79, 368–376. 10.1136/jnnp.2007.131045 18344392

[B77] Jayakar S. S. Dikshit M. (2004). AMPA receptor regulation mechanisms: future target for safer neuroprotective drugs. Int. J. Neurosci. 114, 695–734. 10.1080/00207450490430453 15204061

[B78] Jurado S. (2017). AMPA receptor trafficking in natural and pathological aging. Front. Mol. Neurosci. 10, 446. 10.3389/fnmol.2017.00446 29375307 PMC5767248

[B79] Kandratavicius L. Balista P. A. Lopes-Aguiar C. Ruggiero R. N. Umeoka E. H. Garcia-Cairasco N. (2014). Animal models of epilepsy: use and limitations. Neuropsychiatr. Dis. Treat. 10, 1693–1705. 10.2147/NDT.S50371 25228809 PMC4164293

[B80] Kasteleijn-Nolst Trenite D. Brandt C. Mayer T. Rosenow F. Schmidt B. Steinhoff B. J. (2015). Dose-dependent suppression of human photoparoxysmal response with the competitive AMPA/kainate receptor antagonist BGG492: clear PK/PD relationship. Epilepsia 56, 924–932. 10.1111/epi.13008 25963722

[B81] Kato A. S. Burris K. D. Gardinier K. M. Gernert D. L. Porter W. J. Reel J. (2016). Forebrain-selective AMPA-receptor antagonism guided by TARP gamma-8 as an antiepileptic mechanism. Nat. Med. 22, 1496–1501. 10.1038/nm.4221 27820603

[B82] Kawahara Y. Kwak S. (2005). Excitotoxicity and ALS: what is unique about the AMPA receptors expressed on spinal motor neurons? Amyotroph. Lateral Scler. Other Mot. Neuron Disord. 6, 131–144. 10.1080/14660820510037872 16183555

[B83] Kia A. Mcavoy K. Krishnamurthy K. Trotti D. Pasinelli P. (2018). Astrocytes expressing ALS-linked mutant FUS induce motor neuron death through release of tumor necrosis factor-alpha. Glia 66, 1016–1033. 10.1002/glia.23298 29380416 PMC5873384

[B84] Kim J. E. Choi H. C. Song H. K. Kang T. C. (2019). Perampanel affects up-stream regulatory signaling pathways of GluA1 phosphorylation in normal and epileptic rats. Front. Cell Neurosci. 13, 80. 10.3389/fncel.2019.00080 30881292 PMC6405474

[B85] Klockgether T. Turski L. Honore T. Zhang Z. M. Gash D. M. Kurlan R. (1991). The AMPA receptor antagonist NBQX has antiparkinsonian effects in monoamine-depleted rats and MPTP-treated monkeys. Ann. Neurol. 30, 717–723. 10.1002/ana.410300513 1662477

[B86] Kobylecki C. Cenci M. A. Crossman A. R. Ravenscroft P. (2010). Calciumpermeable AMPA receptors are involved in the induction and expression of l-DOPAinduced dyskinesia in Parkinson’s disease. J. Neurochem. 114, 499–511. 10.1111/j.1471-4159.2010.06776.x 20456008

[B87] Kobylecki C. Crossman A. R. Ravenscroft P. (2013). Alternative splicing of AMPA receptor subunits in the 6-OHDA-lesioned rat model of Parkinson’s disease and L-DOPAinduced dyskinesia. Exp. Neurol. 247, 476–484. 10.1016/j.expneurol.2013.01.019 23360800

[B88] Konitsiotis S. Blanchet P. J. Verhagen L. Lamers E. Chase T. N. (2000). AMPA receptor blockade improves levodopa-induced dyskinesia in MPTP monkeys. Neurology 54, 1589–1595. 10.1212/wnl.54.8.1589 10762498

[B89] Kwon H. S. Koh S. H. (2020). Neuroinflammation in neurodegenerative disorders: the roles of microglia and astrocytes. Transl. Neurodegener. 9, 42. 10.1186/s40035-020-00221-2 33239064 PMC7689983

[B90] Kwong L. K. Uryu K. Trojanowski J. Q. Lee V. M. (2008). TDP-43 proteinopathies: neurodegenerative protein misfolding diseases without amyloidosis. Neurosignals 16, 41–51. 10.1159/000109758 18097159

[B91] Laezza F. Wilding T. J. Sequeira S. Coussen F. Zhang X. Z. Hill-Robinson R. (2007). KRIP6: a novel BTB/kelch protein regulating function of kainate receptors. Mol. Cell Neurosci. 34, 539–550. 10.1016/j.mcn.2006.12.003 17254796 PMC1939939

[B92] Lancaster E. Martinez-Hernandez E. Dalmau J. (2011). Encephalitis and antibodies to synaptic and neuronal cell surface proteins. Neurology 77, 179–189. 10.1212/WNL.0b013e318224afde 21747075 PMC3140073

[B93] Langan Y. M. Lucas R. Jewell H. Toublanc N. Schaefer H. Sander J. W. (2003). Talampanel, a new antiepileptic drug: single- and multiple-dose pharmacokinetics and initial 1-week experience in patients with chronic intractable epilepsy. Epilepsia 44, 46–53. 10.1046/j.1528-1157.2003.128902.x 12581229

[B94] Laryushkin D. P. Maiorov S. A. Zinchenko V. P. Mal'tseva V. N. Gaidin S. G. Kosenkov A. M. (2023). Of the mechanisms of paroxysmal depolarization shifts: generation and maintenance of bicuculline-induced paroxysmal activity in rat hippocampal cell cultures. Int. J. Mol. Sci. 24, 10991. 10.3390/ijms241310991 37446169 PMC10341462

[B95] Lastres-Becker I. Molina-Holgado F. Ramos J. A. Mechoulam R. Fernández-Ruiz J. (2005). Cannabinoids provide neuroprotection against 6-hydroxydopamine toxicity *in vivo* and *in vitro*: relevance to Parkinson’s disease. Neurobiol. Dis. 19, 96–107. 10.1016/j.nbd.2004.11.009 15837565

[B96] Lazarevic V. Yang Y. Ivanova D. Fejtova A. Svenningsson P. (2018). Riluzole attenuates the efficacy of glutamatergic transmission by interfering with the size of the readily releasable neurotransmitter pool. Neuropharmacology 143, 38–48. 10.1016/j.neuropharm.2018.09.021 30222983

[B97] Lees A. Fahn S. Eggert K. M. Jankovic J. Lang A. Micheli F. (2012). Perampanel, an AMPA antagonist, found to have no benefit in reducing "off" time in Parkinson's disease. Mov. Disord. 27, 284–288. 10.1002/mds.23983 22161845

[B98] Leitner D. F. Siu Y. Korman A. Lin Z. Kanshin E. Friedman D. (2023). Metabolomic, proteomic, and transcriptomic changes in adults with epilepsy on modified Atkins diet. Epilepsia 64, 1046–1060. 10.1111/epi.17540 36775798 PMC10372873

[B99] Lim S. N. Wu T. Tseng W. J. Chiang H. I. Cheng M. Y. Lin W. R. (2021). Efficacy and safety of perampanel in refractory and super-refractory status epilepticus: cohort study of 81 patients and literature review. J. Neurol. 268, 3744–3757. 10.1007/s00415-021-10506-9 33754209

[B100] Lin X. J. Zhang J. J. Yu L. C. (2016). GluR2-3Y inhibits the acquisition and reinstatement of morphine-induced conditioned place preference in rats. Neurosci. Bull. 32, 177–182. 10.1007/s12264-016-0018-9 26924808 PMC5563740

[B101] Lindenbach D. Conti M. M. Ostock C. Y. George J. A. Goldenberg A. A. Melikhov-Sosin M. (2016). The role of primary motor cortex (M1) glutamate and GABA signaling in l-DOPA-induced dyskinesia in parkinsonian rats. J. Neurosci. 36, 9873–9887. 10.1523/JNEUROSCI.1318-16.2016 27656025 PMC5030350

[B102] Lippman-Bell J. J. Rakhade S. N. Klein P. M. Obeid M. Jackson M. C. Joseph A. (2013). AMPA receptor antagonist NBQX attenuates later-life epileptic seizures and autistic-like social deficits following neonatal seizures. Epilepsia 54, 1922–1932. 10.1111/epi.12378 24117347 PMC4262152

[B103] Liu S. J. Zukin R. S. (2007). Ca2+-permeable AMPA receptors in synaptic plasticity and neuronal death. Trends Neurosci. 30, 126–134. 10.1016/j.tins.2007.01.006 17275103

[B104] Loscher W. Lehmann H. Behl B. Seemann D. Teschendorf H. J. Hofmann H. P. (1999). A new pyrrolyl-quinoxalinedione series of non-NMDA glutamate receptor antagonists: pharmacological characterization and comparison with NBQX and valproate in the kindling model of epilepsy. Eur. J. Neurosci. 11, 250–262. 10.1046/j.1460-9568.1999.00432.x 9987029

[B105] Lourenco J. Cannich A. Carta M. Coussen F. Mulle C. Marsicano G. (2010). Synaptic activation of kainate receptors gates presynaptic CB(1) signaling at GABAergic synapses. Nat. Neurosci. 13, 197–204. 10.1038/nn.2481 20081851

[B106] Lourenco J. Matias I. Marsicano G. Mulle C. (2011). Pharmacological activation of kainate receptors drives endocannabinoid mobilization. J. Neurosci. 31, 3243–3248. 10.1523/JNEUROSCI.3512-10.2011 21368036 PMC6623943

[B107] Lutsenko V. K. Vukolova M. N. Kucheryanu V. G. Gudasheva T. A. (2003). Dipeptide analog of neurotensin active site prevents the development of experimental Parkinson’s syndrome in mice. Bull. Exp. Biol. Med. 136, 352–354. 10.1023/b:bebm.0000010949.44563.15 14714080

[B108] Lykens N. M. Coughlin D. J. Reddi J. M. Lutz G. J. Tallent M. K. (2017). AMPA GluA1-flip targeted oligonucleotide therapy reduces neonatal seizures and hyperexcitability. PLoS One 12, e0171538. 10.1371/journal.pone.0171538 28178321 PMC5298276

[B109] Lynch G. (2006). Glutamate-based therapeutic approaches: ampakines. Curr. Opin. Pharmacol. 6, 82–88. 10.1016/j.coph.2005.09.005 16361116

[B110] Maher M. P. Wu N. Ravula S. Ameriks M. K. Savall B. M. Liu C. (2016). Discovery and characterization of AMPA receptor modulators selective for TARP-γ8. J. Pharmacol. Exp. Ther. 357, 394–414. 10.1124/jpet.115.231712 26989142

[B111] Maiti P. Manna J. Dunbar G. L. (2017). Current understanding of the molecular mechanisms in Parkinson’s disease: targets for potential treatments. Transl. Neurodegener. 6, 28. 10.1186/s40035-017-0099-z 29090092 PMC5655877

[B112] Malek N. (2019). Deep brain stimulation in Parkinson’s disease. Neurol. India 67, 968–978. 10.4103/0028-3886.266268 31512617

[B113] Mammi A. Ferlazzo E. Gasparini S. Bova V. Neri S. Labate A. (2022). Psychiatric and behavioural side effects associated with perampanel in patients with temporal lobe epilepsy. A real-world experience. Front. Neurol. 13, 839985. 10.3389/fneur.2022.839985 35321512 PMC8936072

[B114] Maraschi A. Ciammola A. Folci A. Sassone F. Ronzitti G. Cappelletti G. (2014). Parkin regulates kainate receptors by interacting with the GluK2 subunit. Nat. Commun. 5, 5182. 10.1038/ncomms6182 25316086 PMC4218952

[B115] Marin C. Jimenez A. Bonastre M. Vila M. Agid Y. Hirsch E. C. (2001). LY293558, an AMPA glutamate receptor antagonist, prevents and reverses levodopa-induced motor alterations in Parkinsonian rats. Synapse 42, 40–47. 10.1002/syn.1097 11668589

[B116] Marshall J. J. Xu J. Contractor A. (2018). Kainate receptors inhibit glutamate release via mobilization of endocannabinoids in striatal direct pathway spiny projection neurons. J. Neurosci. 38, 3901–3910. 10.1523/JNEUROSCI.1788-17.2018 29540547 PMC5907053

[B117] Martin-Mcgill K. J. Bresnahan R. Levy R. G. Cooper P. N. (2020). Ketogenic diets for drug-resistant epilepsy. Cochrane Database Syst. Rev. 6, CD001903. 10.1002/14651858.CD001903.pub5 32588435 PMC7387249

[B118] Martin-Moreno A. M. Reigada D. Ramirez B. G. Mechoulam R. Innamorato N. Cuadrado A. (2011). Cannabidiol and other cannabinoids reduce microglial activation *in vitro* and *in vivo*: relevance to Alzheimer’s disease. Mol. Pharmacol. 79, 964–973. 10.1124/mol.111.071290 21350020 PMC3102548

[B119] Mayer M. L. Westbrook G. L. Guthrie P. B. (1984). Voltage-dependent block by Mg2+ of NMDA responses in spinal cord neurones. Nature 309, 261–263. 10.1038/309261a0 6325946

[B120] Menon P. Kiernan M. C. Vucic S. (2014). Biomarkers and future targets for development in amyotrophic lateral sclerosis. Curr. Med. Chem. 21, 3535–3550. 10.2174/0929867321666140601161148 24934359

[B121] Mironova Y. S. Zhukova I. A. Zhukova N. G. Alifirova V. M. Izhboldina O. P. Latypova A. V. (2018). Parkinson’s disease and glutamate excitotoxicity. Zh Nevrol. Psikhiatr Im. S S Korsakova 118, 50–54. 10.17116/jnevro201811806250 30346434

[B122] Moodley K. K. Chan D. (2014). The hippocampus in neurodegenerative disease. Front. Neurol. Neurosci. 34, 95–108. 10.1159/000356430 24777134

[B123] Moshe S. L. Perucca E. Ryvlin P. Tomson T. (2015). Epilepsy: new advances. Lancet 385, 884–898. 10.1016/S0140-6736(14)60456-6 25260236

[B124] Murray T. K. Whalley K. Robinson C. S. Ward M. A. Hicks C. A. Lodge D. (2003). LY503430, a novel alpha-amino-3-hydroxy-5-methylisoxazole-4-propionic acid receptor potentiator with functional, neuroprotective and neurotrophic effects in rodent models of Parkinson’s disease. J. Pharmacol. Exp. Ther. 306, 752–762. 10.1124/jpet.103.049445 12730350

[B125] Naziroglu M. (2015). TRPV1 channel: a potential drug target for treating epilepsy. Curr. Neuropharmacol. 13, 239–247. 10.2174/1570159x13666150216222543 26411767 PMC4598436

[B126] Neumann M. Sampathu D. M. Kwong L. K. Truax A. C. Micsenyi M. C. Chou T. T. (2006). Ubiquitinated TDP-43 in frontotemporal lobar degeneration and amyotrophic lateral sclerosis. Science 314, 130–133. 10.1126/science.1134108 17023659

[B127] Nowak L. Bregestovski P. Ascher P. Herbet A. Prochiantz A. (1984). Magnesium gates glutamate-activated channels in mouse central neurones. Nature 307, 462–465. 10.1038/307462a0 6320006

[B128] Olney J. W. (1994). Excitatory transmitter neurotoxicity. Neurobiol. Aging 15, 259–260. 10.1016/0197-4580(94)90127-9 7838306

[B129] O’Neill M. J. Bleakman D. Zimmerman D. M. Nisenbaum E. S. (2004). AMPA receptor potentiators for the treatment of CNS disorders. Curr. Drug Targets CNS Neurol. Disord. 3, 181–194. 10.2174/1568007043337508 15180479

[B130] O’Neill M. J. Murray T. K. Clay M. P. Lindstrom T. Yang C. R. Nisenbaum E. S. (2005). LY503430: pharmacology, pharmacokinetics, and effects in rodent models of Parkinson’s disease. CNS Drug Rev. 11, 77–96. 10.1111/j.1527-3458.2005.tb00037.x 15867954 PMC6741716

[B131] O’Neill M. J. Witkin J. M. (2007). AMPA receptor potentiators: application for depression and Parkinson’s disease. Curr. Drug Targets 8, 603–620. 10.2174/138945007780618517 17504104

[B132] Orain D. Tasdelen E. Haessig S. Koller M. Picard A. Dubois C. (2017). Design and synthesis of selurampanel, a novel orally active and competitive AMPA receptor antagonist. Chem. Med. Chem. 12, 197–201. 10.1002/cmdc.201600467 27863026

[B133] Oskarsson B. Mauricio E. A. Shah J. S. Li Z. Rogawski M. A. (2021). Cortical excitability threshold can be increased by the AMPA blocker Perampanel in amyotrophic lateral sclerosis. Muscle Nerve 64, 215–219. 10.1002/mus.27328 34008857

[B134] Ossowska K. (1994). The role of excitatory amino acids in experimental models of Parkinson’s disease. J. Neural Transm. Park Dis. Dement. Sect. 8, 39–71. 10.1007/BF02250917 7534462

[B135] Pacher P. Kogan N. M. Mechoulam R. (2020). Beyond THC and endocannabinoids. Annu. Rev. Pharmacol. Toxicol. 60, 637–659. 10.1146/annurev-pharmtox-010818-021441 31580774

[B136] Pagano C. Navarra G. Coppola L. Avilia G. Bifulco M. Laezza C. (2022). Cannabinoids: therapeutic use in clinical practice. Int. J. Mol. Sci. 23, 3344. 10.3390/ijms23063344 35328765 PMC8952215

[B137] Paizs M. Tortarolo M. Bendotti C. Engelhardt J. I. Siklos L. (2011). Talampanel reduces the level of motoneuronal calcium in transgenic mutant SOD1 mice only if applied presymptomatically. Amyotroph. Lateral. Scler. 12, 340–344. 10.3109/17482968.2011.584627 21623665 PMC3231880

[B138] Paoletti P. Bellone C. Zhou Q. (2013). NMDA receptor subunit diversity: impact on receptor properties, synaptic plasticity and disease. Nat. Rev. Neurosci. 14, 383–400. 10.1038/nrn3504 23686171

[B139] Papa S. M. Chase T. N. (1996). Levodopa‐induced dyskinesias improved by a glutamate antagonist in parkinsonia monkeys. Ann. Neurol. 39, 574–578. 10.1002/ana.410390505 8619541

[B140] Parsons M. P. Raymond L. A. (2014). Extrasynaptic NMDA receptor involvement in central nervous system disorders. Neuron 82, 279–293. 10.1016/j.neuron.2014.03.030 24742457

[B141] Partin K. M. (2015). AMPA receptor potentiators: from drug design to cognitive enhancement. Curr. Opin. Pharmacol. 20, 46–53. 10.1016/j.coph.2014.11.002 25462292 PMC4318786

[B142] Pascuzzi R. M. Shefner J. Chappell A. S. Bjerke J. S. Tamura R. Chaudhry V. (2010). A phase II trial of talampanel in subjects with amyotrophic lateral sclerosis. Amyotroph. Lateral Scler. 11, 266–271. 10.3109/17482960903307805 19961264

[B143] Patai R. Paizs M. Tortarolo M. Bendotti C. Obal I. Engelhardt J. I. (2017). Presymptomatically applied AMPA receptor antagonist prevents calcium increase in vulnerable type of motor axon terminals of mice modeling amyotrophic lateral sclerosis. Biochim. Biophys. Acta Mol. Basis Dis. 1863, 1739–1748. 10.1016/j.bbadis.2017.05.016 28528135

[B144] Patra P. H. Barker-Haliski M. White H. S. Whalley B. J. Glyn S. Sandhu H. (2019). Cannabidiol reduces seizures and associated behavioral comorbidities in a range of animal seizure and epilepsy models. Epilepsia 60, 303–314. 10.1111/epi.14629 30588604 PMC6378611

[B145] Peres F. F. Lima A. C. Hallak J. E. C. Crippa J. A. Silva R. H. Abilio V. C. (2018). Cannabidiol as a promising strategy to treat and prevent movement disorders? Front. Pharmacol. 9, 482. 10.3389/fphar.2018.00482 29867488 PMC5958190

[B146] Perier C. Marin C. Bonastre M. Tolosa E. Hirsch E. C. (2002). AMPA receptor antagonist LY293558 reverses preproenkephalin mRNA overexpression in the striatum of 6-OHDA-lesioned-rats treated with L-dopa. Eur. J. Neurosci. 16, 2236–2240. 10.1046/j.1460-9568.2002.02275.x 12473092

[B147] Pinheiro P. S. Lanore F. Veran J. Artinian J. Blanchet C. Crepel V. (2013). Selective block of postsynaptic kainate receptors reveals their function at hippocampal mossy fiber synapses. Cereb. Cortex. 23, 323–331. 10.1093/cercor/bhs022 22345355

[B148] Pitkänen A. Mathiesen C. Rønn L. C. Møller A. Nissinen J. (2007). Effect of novel AMPA antagonist, NS1209, on status epilepticus. Epilepsy Res. 74, 45–54. 10.1016/j.eplepsyres.2006.12.004 17289347

[B149] Post B. Van Den Heuvel L. Van Prooije T. Van Ruissen X. Van De Warrenburg B. Nonnekes J. (2020). Young onset Parkinson’s disease: a modern and tailored approach. J. Park. Dis. 10, S29–S36. 10.3233/JPD-202135 PMC7592661 32651336

[B150] Potschka H. Trinka E. (2019). Perampanel: does it have broad-spectrum potential? Epilepsia 60, 22–36. 10.1111/epi.14456 29953584

[B151] Ramsdell J. S. Gulland F. M. (2014). Domoic acid epileptic disease. Mar. Drugs 12, 1185–1207. 10.3390/md12031185 24663110 PMC3967204

[B152] Rascol O. Barone P. Behari M. Emre M. Giladi N. Olanow C. W. (2012). Perampanel in Parkinson disease fluctuations: a double-blind randomized trial with placebo and entacapone. Clin. Neuropharmacol. 35, 15–20. 10.1097/WNF.0b013e318241520b 22222634

[B153] Re D. B. Le Verche V. Yu C. Amoroso M. W. Politi K. A. Phani S. (2014). Necroptosis drives motor neuron death in models of both sporadic and familial ALS. Neuron 81, 1001–1008. 10.1016/j.neuron.2014.01.011 24508385 PMC3951532

[B154] Regoni M. Cattaneo S. Mercatelli D. Novello S. Passoni A. Bagnati R. (2020). Pharmacological antagonism of kainate receptor rescues dysfunction and loss of dopamine neurons in a mouse model of human parkin-induced toxicity. Cell Death Dis. 11, 963. 10.1038/s41419-020-03172-8 33173027 PMC7656261

[B155] Rogawski M. A. (2013). AMPA receptors as a molecular target in epilepsy therapy. Acta Neurol. Scand. Suppl, 9–18. 10.1111/ane.12099 PMC4506648 23480151

[B156] Rogawski M. A. Kurzman P. S. Yamaguchi S. I. Li H. (2001). Role of AMPA and GluR5 kainate receptors in the development and expression of amygdala kindling in the mouse. Neuropharmacology 40, 28–35. 10.1016/s0028-3908(00)00112-x 11077068

[B157] Rogawski M. A. Le D. Q. Uyakul D. Pannell L. K. Subramaniam S. Yamaguchi S. (1995). Anticonvulsant efficacy of ADCI (5-aminocarbonyl-10,11-dihydro-5Hdibenzo[a,d]cyclohepten-5,10-imine) after acute and chronic dosing in mice. Epilepsia 36, 566–571. 10.1111/j.1528-1157.1995.tb02568.x 7555968

[B158] Roisen F. J. Bartfeld H. Donnenfeld H. Baxter J. (1982). Neuron specific *in vitro* cytotoxicity of sera from patients with amyotrophic lateral sclerosis. Muscle Nerve 5, 48–53. 10.1002/mus.880050109 7057805

[B159] Rosen D. R. Siddique T. Patterson D. Figlewicz D. A. Sapp P. Hentati A. (1993). Mutations in Cu/Zn superoxide dismutase gene are associated with familial amyotrophic lateral sclerosis. Nature 362, 59–62. 10.1038/362059a0 8446170

[B160] Rosenblum L. T. Trotti D. (2017). EAAT2 and the molecular signature of amyotrophic lateral sclerosis. Adv. Neurobiol. 16, 117–136. 10.1007/978-3-319-55769-4_6 28828608 PMC6668619

[B161] Rothstein J. D. Tsai G. Kuncl R. W. Clawson L. Cornblath D. R. Drachman D. B. (1990). Abnormal excitatory amino acid metabolism in amyotrophic lateral sclerosis. Ann. Neurol. 28, 18–25. 10.1002/ana.410280106 2375630

[B162] Ruel J. Guitton M. J. Puell J. L. (2002). Negative allosteric modulation of AMPA-preferring receptors by the selective isomer GYKI 53784 (LY303070), a specific non-competitive AMPA antagonist. CNS Drug Rev. 8, 235–254. 10.1111/j.1527-3458.2002.tb00227.x 12353057 PMC6741693

[B163] Ruiz-Valdepenas L. Martinez-Orgado J. A. Benito C. Millan A. Tolon R. M. Romero J. (2011). Cannabidiol reduces lipopolysaccharide-induced vascular changes and inflammation in the mouse brain: an intravital microscopy study. J. Neuroinflammation 8, 5. 10.1186/1742-2094-8-5 21244691 PMC3034694

[B164] Sabers A. Wolf P. Moller A. Rysgaard K. Ben-Menachem E. (2013). A prospective, randomized, multicentre trial for the treatment of refractory status epilepticus; experiences from evaluating the effect of the novel drug candidate, NS1209. Epilepsy Res. 106, 292–295. 10.1016/j.eplepsyres.2013.04.002 23623849

[B165] Salpietro V. Dixon C. L. Guo H. Bello O. D. Vandrovcova J. Efthymiou S. (2019). AMPA receptor GluA2 subunit defects are a cause of neurodevelopmental disorders. Nat. Commun. 10, 3094. 10.1038/s41467-019-10910-w 31300657 PMC6626132

[B166] Sarlo G. L. Holton K. F. (2021). Brain concentrations of glutamate and GABA in human epilepsy: a review. Seizure 91, 213–227. 10.1016/j.seizure.2021.06.028 34233236

[B167] Shaw P. J. Forrest V. Ince P. G. Richardson J. P. Wastell H. J. (1995). CSF and plasma amino acid levels in motor neuron disease: elevation of CSF glutamate in a subset of patients. Neurodegeneration 4, 209–216. 10.1006/neur.1995.0026 7583686

[B168] Sills M. A. Forsythe W. I. Haidukewych D. Macdonald A. Robinson M. (1986). The medium chain triglyceride diet and intractable epilepsy. Arch. Dis. Child. 61, 1168–1172. 10.1136/adc.61.12.1168 3101615 PMC1778211

[B169] Silverdale M. A. Kobylecki C. Hallett P. J. Li Q. Dunah A. W. Ravenscroft P. (2010). Synaptic recruitment of AMPA glutamate receptor subunits in levodopainduced dyskinesia in the MPTP-lesioned nonhuman primate. Synapse 64, 177–180. 10.1002/syn.20739 19852073

[B170] Smolders I. Bortolotto Z. A. Clarke V. R. Warre R. Khan G. M. O'Neill M. J. (2002). Antagonists of GLU(K5)-containing kainate receptors prevent pilocarpine-induced limbic seizures. Nat. Neurosci. 5, 796–804. 10.1038/nn880 12080343

[B171] Sobolevsky A. I. Yelshansky M. V. (2000). The trapping block of NMDA receptor channels in acutely isolated rat hippocampal neurones. J. Physiol. 526, 493–506. 10.1111/j.1469-7793.2000.t01-2-00493.x 10922002 PMC2270033

[B172] Stauch Slusher B. Rissolo K. C. Anzilotti K. F. JR. Jackson P. F. (1995). Centrally-administered AMPA antagonists increase locomotion in parkinsonian rats. J. Neural Transm. Park Dis. Dement. Sect. 9, 145–149. 10.1007/BF02259656 8526999

[B173] Stayte S. Laloli K. J. Rentsch P. Lowth A. Li K. M. Pickford R. (2020). The kainate receptor antagonist UBP310 but not single deletion of GluK1, GluK2, or GluK3 subunits, inhibits MPTP-induced degeneration in the mouse midbrain. Exp. Neurol. 323, 113062. 10.1016/j.expneurol.2019.113062 31513786

[B174] Steinhoff B. J. Ben-Menachem E. Ryvlin P. Shorvon S. Kramer L. Satlin A. (2013). Efficacy and safety of adjunctive perampanel for the treatment of refractory partial seizures: a pooled analysis of three phase III studies. Epilepsia 54, 1481–1489. 10.1111/epi.12212 23663001

[B175] Tamano H. Morioka H. Nishio R. Takeuchi A. Takeda A. (2018). AMPA-induced extracellular Zn^2+^ influx into nigral dopaminergic neurons causes movement disorder in rats. Neurotoxicology 69, 23–28. 10.1016/j.neuro.2018.08.008 30176255

[B176] Taniguchi S. Stolz J. R. Swanson G. T. (2022). The antiseizure drug perampanel is a subunit-selective negative allosteric modulator of kainate receptors. J. Neurosci. 42, 5499–5509. 10.1523/JNEUROSCI.2397-21.2022 35654603 PMC9295835

[B177] Tanner C. M. Kamel F. Ross G. W. Hoppin J. A. Goldman S. M. Korell M. (2011). Rotenone, paraquat, and Parkinson’s disease. Environ. Health Perspect. 119, 866–872. 10.1289/ehp.1002839 21269927 PMC3114824

[B178] Tarantino N. Canfora I. Camerino G. M. Pierno S. (2022). Therapeutic targets in amyotrophic lateral sclerosis: focus on ion channels and skeletal muscle. Cells 11, 415. 10.3390/cells11030415 35159225 PMC8834084

[B179] Taylor J. P. Brown R. H. Cleveland D. W. (2016). Decoding ALS: from genes to mechanism. Nature 539, 197–206. 10.1038/nature20413 27830784 PMC5585017

[B180] Tolosa E. Garrido A. Scholz S. W. Poewe W. (2021). Challenges in the diagnosis of Parkinson’s disease. Lancet Neurol. 20, 385–397. 10.1016/S1474-4422(21)00030-2 33894193 PMC8185633

[B181] Traynelis S. F. Wollmuth L. P. Mcbain C. J. Menniti F. S. Vance K. M. Ogden K. K. (2010). Glutamate receptor ion channels: structure, regulation, and function. Pharmacol. Rev. 62, 405–496. 10.1124/pr.109.002451 20716669 PMC2964903

[B182] Twele F. Bankstahl M. Klein S. Romermann K. Loscher W. (2015). The AMPA receptor antagonist NBQX exerts anti-seizure but not antiepileptogenic effects in the intrahippocampal kainate mouse model of mesial temporal lobe epilepsy. Neuropharmacology 95, 234–242. 10.1016/j.neuropharm.2015.03.014 25839899

[B183] Twomey E. C. Yelshanskaya M. V. Sobolevsky A. I. (2019). Structural and functional insights into transmembrane AMPA receptor regulatory protein complexes. J. Gen. Physiol. 151, 1347–1356. 10.1085/jgp.201812264 31615831 PMC6888759

[B184] Ueda J. Uemura N. Sawamura M. Taguchi T. Ikuno M. Kaji S. Y. (2021). Perampanel inhibits α-synuclein transmission in Parkinson's disease models. Mov. Disord. 36, 1554–1564. 10.1002/mds.28558 33813737

[B185] Van Damme P. Leyssen M. Callewaert G. Robberecht W. Van Den Bosch L. (2003). The AMPA receptor antagonist NBQX prolongs survival in a transgenic mouse model of amyotrophic lateral sclerosis. Neurosci. Lett. 343, 81–84. 10.1016/s0304-3940(03)00314-8 12759169

[B186] Van Den Bos M. A. J. Geevasinga N. Higashihara M. Menon P. Vucic S. (2019). Pathophysiology and diagnosis of ALS: insights from advances in neurophysiological techniques. Int. J. Mol. Sci. 20, 2818. 10.3390/ijms20112818 31185581 PMC6600525

[B187] Van Den Bosch L. Vandenberghe W. Klaassen H. Van Houtte E. Robberecht W. (2000). Ca(2+)-permeable AMPA receptors and selective vulnerability of motor neurons. J. Neurol. Sci. 180, 29–34. 10.1016/s0022-510x(00)00414-7 11090861

[B188] Vargas J. R. Takahashi D. K. Thomson K. E. Wilcox K. S. (2013). The expression of kainate receptor subunits in hippocampal astrocytes after experimentally induced status epilepticus. J. Neuropathol. Exp. Neurol. 72, 919–932. 10.1097/NEN.0b013e3182a4b266 24042195 PMC3880830

[B189] Wang J. Wang F. Mai D. Qu S. (2020). Molecular mechanisms of glutamate toxicity in Parkinson’s disease. Front. Neurosci. 14, 585584. 10.3389/fnins.2020.585584 33324150 PMC7725716

[B190] Wang L. Gutmann D. H. Roos R. P. (2011). Astrocyte loss of mutant SOD1 delays ALS disease onset and progression in G85R transgenic mice. Hum. Mol. Genet. 20, 286–293. 10.1093/hmg/ddq463 20962037

[B191] Wyeth M. S. Pelkey K. A. Yuan X. Vargish G. Johnston A. D. Hunt S. (2017). Neto auxiliary subunits regulate interneuron somatodendritic and presynaptic kainate receptors to control network inhibition. Cell Rep. 20, 2156–2168. 10.1016/j.celrep.2017.08.017 28854365 PMC5600503

[B192] Yamanaka K. Chun S. J. Boillee S. Fujimori-Tonou N. Yamashita H. Gutmann D. H. (2008). Astrocytes as determinants of disease progression in inherited amyotrophic lateral sclerosis. Nat. Neurosci. 11, 251–253. 10.1038/nn2047 18246065 PMC3137510

[B193] Yamashita T. Kwak S. (2019). Cell death cascade and molecular therapy in ADAR2-deficient motor neurons of ALS. Neurosci. Res. 144, 4–13. 10.1016/j.neures.2018.06.004 29944911

[B194] Yelshanskaya M. V. Singh A. K. Narangoda C. Williams R. S. B. Kurnikova M. G. Sobolevsky A. I. (2022). Structural basis of AMPA receptor inhibition by trans4-butylcyclohexane carboxylic acid. Br. J. Pharmacol. 179, 3628–3644. 10.1111/bph.15254 32959886 PMC10693435

[B195] Yelshanskaya M. V. Singh A. K. Sampson J. M. Narangoda C. Kurnikova M. Sobolevsky A. I. (2016a). Structural bases of noncompetitive inhibition of AMPA-subtype ionotropic glutamate receptors by antiepileptic drugs. Neuron 91, 1305–1315. 10.1016/j.neuron.2016.08.012 27618672 PMC5033713

[B196] Yelshanskaya M. V. Sobolevsky A. I. (2022). Structural insights into function of ionotropic glutamate receptors. Biochem. Mosc. Suppl. Ser. A Membr. Cell Biol. 16, 190–206. 10.1134/s1990747822040043 PMC12860490 41625315

[B197] Yin H. Z. Hsu C. I. Yu S. Rao S. D. Sorkin L. S. Weiss J. H. (2012). TNF-alpha triggers rapid membrane insertion of Ca(2+) permeable AMPA receptors into adult motor neurons and enhances their susceptibility to slow excitotoxic injury. Exp. Neurol. 238, 93–102. 10.1016/j.expneurol.2012.08.004 22921461 PMC3498614

[B198] Yu Y. Yang Z. Jin B. Qin X. Zhu X. Sun J. (2020). Cannabidiol inhibits febrile seizure by modulating AMPA receptor kinetics through its interaction with the N-terminal domain of GluA1/GluA2. Pharmacol. Res. 161, 105128. 10.1016/j.phrs.2020.105128 32805354

[B199] Zhang H. Bramham C. R. (2020). Bidirectional dysregulation of AMPA receptor-mediated synaptic transmission and plasticity in brain disorders. Front. Synaptic Neurosci. 12, 26. 10.3389/fnsyn.2020.00026 32754026 PMC7366028

[B200] Zhang J. Abdullah J. M. (2013). The role of GluA1 in central nervous system disorders. Rev. Neurosci. 24, 499–505. 10.1515/revneuro-2013-0021 24077616

[B201] Zhang J. Lv S. Tang G. Bian G. Yang Y. Li R. (2019a). Activation of calcium-impermeable GluR2-containing AMPA receptors in the lateral habenula produces antidepressant-like effects in a rodent model of Parkinson’s disease. Exp. Neurol. 322, 113058. 10.1016/j.expneurol.2019.113058 31499061

[B202] Zhang J. Wang Y. Sun Y. N. Li L. B. Zhang L. Guo Y. (2019b). Blockade of calcium-permeable AMPA receptors in the lateral habenula produces increased antidepressant-like effects in unilateral 6-hydroxydopamine-lesioned rats compared to sham-lesioned rats. Neuropharmacology 157, 107687. 10.1016/j.neuropharm.2019.107687 31251995

[B203] Zhang Z. Zhang S. Fu P. Zhang Z. Lin K. Ko J. K. (2019c). Roles of glutamate receptors in Parkinson’s disease. Int. J. Mol. Sci. 20, 4391. 10.3390/ijms20184391 31500132 PMC6769661

[B204] Zhao P. Leonoudakis D. Abood M. E. Beattie E. C. (2010). Cannabinoid receptor activation reduces TNF-alpha-induced surface localization of AMPAR-type glutamate receptors and excitotoxicity. Neuropharmacology 58, 551–558. 10.1016/j.neuropharm.2009.07.035 19654014 PMC3951320

[B205] Zuddas A. Oberto G. Vaglini F. Fascetti F. Fornai F. Corsini G. U. (1992). MK-801 prevents 1-methyl-4-phenyl-1,2,3,6-tetrahydropyridine-induced parkinsonism in primates. J. Neurochem. 59, 733–739. 10.1111/j.1471-4159.1992.tb09429.x 1629743

